# Tumour-suppressor microRNAs regulate ovarian cancer cell physical properties and invasive behaviour

**DOI:** 10.1098/rsob.160275

**Published:** 2016-11-30

**Authors:** Clara K. Chan, Yinghong Pan, Kendra Nyberg, Marco A. Marra, Emilia L. Lim, Steven J. M. Jones, Dianna Maar, Ewan A. Gibb, Preethi H. Gunaratne, A. Gordon Robertson, Amy C. Rowat

**Affiliations:** 1 Department of Integrative Biology and Physiology, University of California Los Angeles, Los Angeles, CA, USA; 2 Department of Bioengineering, University of California Los Angeles, Los Angeles, CA, USA; 3 Department of Biochemistry and Biology, University of Houston, Houston, TX, USA; 4 Department of Pathology and Immunology, Baylor College of Medicine, Houston, TX, USA; 5 Human Genome Sequencing Center, Baylor College of Medicine, Houston, TX, USA; 6 British Columbia Cancer Agency, Canada's Michael Smith Genome Sciences Centre, Vancouver, British Columbia, Canada; 7 Department of Medical Genetics, University of British Columbia, Vancouver, British Columbia, Canada; 8 Department of Molecular Biology and Biochemistry, Simon Fraser University, Burnaby, British Columbia, Canada; 9 Bio-Rad Laboratories, The Digital Biology Center, Pleasanton, CA, USA; 10 Jonsson Comprehensive Cancer Center, University of California Los Angeles, Los Angeles, CA, USA

**Keywords:** cell deformability, actin cytoskeleton, microfluidics, microfiltration, tumour cell invasion

## Abstract

The activities of pathways that regulate malignant transformation can be influenced by microRNAs (miRs). Recently, we showed that increased expression of five tumour-suppressor miRs, miR-508-3p, miR-508-5p, miR-509-3p, miR-509-5p and miR-130b-3p, correlate with improved clinical outcomes in human ovarian cancer patients, and that miR-509-3p attenuates invasion of ovarian cancer cell lines. Here, we investigate the mechanism underlying this reduced invasive potential by assessing the impact of these five miRs on the physical properties of cells. Human ovarian cancer cells (HEYA8, OVCAR8) that are transfected with miR mimics representing these five miRs exhibit decreased invasion through collagen matrices, increased cell size and reduced deformability as measured by microfiltration and microfluidic assays. To understand the molecular basis of altered invasion and deformability induced by these miRs, we use predicted and validated mRNA targets that encode structural and signalling proteins that regulate cell mechanical properties. Combined with analysis of gene transcripts by real-time PCR and image analysis of F-actin in single cells, our results suggest that these tumour-suppressor miRs may alter cell physical properties by regulating the actin cytoskeleton. Our findings provide biophysical insights into how tumour-suppressor miRs can regulate the invasive behaviour of ovarian cancer cells, and identify potential therapeutic targets that may be implicated in ovarian cancer progression.

## 1. Introduction

High-grade serous ovarian cancer (HGSC) is the most lethal gynaecological cancer in the USA [1]. Most patients initially respond to platinum and taxane-based treatments, but subsequently develop chemoresistance; the 5-year survival rate is only 44% [1]. The critical need for successful treatment strategies motivated The Cancer Genome Atlas (TCGA) Network to catalogue the genetic alterations in approximately 500 HGSC samples [2]. Through computational analysis of TCGA data, we identified a panel of differentially expressed microRNA (miR) mature strands (miR-508-3p, miR-508-5p, miR-509-3p, miR-509-5p and miR-130b-3p), for which higher levels of expression are associated with longer survival in HGSC patients [3]. Improved survival can be associated with reduced metastasis and decreased invasion of cancer cells. Our previous work shows that overexpression of tumour-suppressor miR-509-3p in human ovarian cancer cell lines impedes invasion through Matrigel matrices [3]. Furthermore, downregulation of miR-130b-3p is associated with cancer progression and multidrug resistance in ovarian cancer [4]. While miRs such as miR-509-3p impact proliferation and cell signalling behaviour [5,6], which could influence metastatic dissemination and growth of cancer cells, the invasion and migration of cancer cells from a primary tumour involves physical and mechanical processes. Detailed biophysical investigations at the single-cell level could thus deepen our understanding of how elevated levels of tumour-suppressor miRs may result in clinical benefits.

During cancer progression, tumour cells undergo changes in their physical properties, including their deformability and adhesion, which can contribute to their altered motility and invasive behaviour [7–9]. Such physical properties are relevant for understanding how cells deform through narrow gaps in the circulatory system, which is required for metastasis [10]; they are also essential for invasion through the extracellular matrix (ECM), connective tissue and endothelial linings of blood or lymph vessels [11]. The ECM is composed of glycoproteins, proteoglycans and fibrous proteins such as collagens [12], which form a network with gaps or pores that range in diameter from 10 nm to 5 µm [13–15]; increased cell and nuclear volume is correlated with reduced invasion of breast cancer cells through *in vitro* collagen gels [16]. To overcome the physical constraints imposed by ECM barriers, cells secrete proteases, such as matrix metalloproteases (MMPs), which can increase the size of gaps between neighbouring fibres [17–19]. Many types of tumour cells are also more deformable compared with benign cells [20–22], and cell mechanical properties are associated with invasion efficiency [16,23,24]. Compared with less deformable ovarian tumour cells that have a higher Young's modulus or decreased compliance, cancer cells that are more deformable tend to move more quickly through the gaps of *in vitro* transwell migration and invasion assays [23,24]. Considering the large deformations required during extra- and intravasation as well as invasion into surrounding tissues, changes in the size and deformability of single tumour cells could play a functional role in disease progression. We hypothesize that altered cell physical properties may reduce cell invasion, and thereby contribute to the improved prognosis, which is associated with higher levels of tumour-suppressor miRs.

To determine the effect of tumour-suppressor miRs on cancer cell physical properties, we overexpress a panel of five miRs (miR-508-3p, miR-508-5p, miR-509-3p, miR-509-5p and miR-130b-3p) in human ovarian carcinoma cells (HEYA8, OVCAR8) using miR mimics for each. We characterize the ability of cells to invade through collagen matrices in the presence of an MMP inhibitor; the inhibitor limits matrix degradation and enhances the extent to which cells must deform to move through the steric constraints of collagen gels. To determine cell deformability, we drive cells to passively deform through micrometre-scale pores using microfluidic deformation [25,26] and parallel microfiltration (PMF) [27] assays. To gain insight into the molecular basis of the effects of tumour-suppressor miRs on cell physical properties, we identify predicted miR–mRNA targets that encode structural or signalling proteins that regulate cell mechanical properties; we also verify transcript levels of selected predicted targets. Through analysis of miR–mRNA interactions, our results show that these tumour-suppressor miRs are predicted to target genes that are implicated in the structure and remodelling of the actin cytoskeleton. By imaging cells in both suspended and adhered states using imaging flow cytometry and confocal microscopy, we observe increased levels of filamentous actin (F-actin) with miR overexpression, and a strong inverse correlation between invasive potential and F-actin levels in adhered cells. Taken together, our results reveal that these five tumour-suppressor miRs that reduce cell invasive behaviour are implicated in the structure and remodelling of the actin cytoskeleton. Our findings also identify novel proteins for future study that may potentially serve as new druggable targets that play a role in ovarian cancer cell invasion and disease progression.

## 2. Material and methods

### 2.1. Cell culture and transfection

Ovarian cancer cells (HEYA8, OVCAR8) are cultured in RPMI 1640 medium supplemented with 10% heat-inactivated fetal bovine serum (FBS) and 1% of penicillin/streptomycin. Cells are grown under standard conditions at 37°C and 5% CO_2_. MiR mimics and scrambled (SCR) negative controls are transiently transfected at 24 nM using Lipofectamine 2000 in serum-free OptiMEM medium, followed by the addition of 10% FBS after 4 h in serum-free conditions. All assays are performed 72 h post-transfection.

### 2.2. Scratch wound invasion assay

To measure cell invasive potential, cells are seeded on 30 µg ml^−1^ collagen-coated 96-well microplates at a density of 27 000 cells per well. After overnight culture at 37°C with 5% CO_2_, scratch wounds are generated on the confluent cell monolayer using sterile P1000 pipette tips and washed with PBS to remove debris and to prevent dislodged cells from settling and reattaching. We prepare collagen gels on ice immediately prior to use by diluting collagen type I (rat tail collagen I, Corning) in RPMI 1640 medium with 50 µM of the MMP inhibitor GM6001 (Santa Cruz Biotechnology), to a final concentration of 1 mg ml^−1^ collagen; 0.1 M NaOH is added to bring the pH to 7.4. Thereafter, 50 μl of collagen solution is added to each well and incubated at 37°C for 1 h, followed by the addition of 200 μl of culture medium with 50 µM GM6001. To confirm reduced MMP activity, we measure total protease activity using 25 µl of 20 µM fluorogenic peptide substrate in 75 µl of conditioned media after 72 h of cell culture (ES010, R&D Systems), followed by a 30 min incubation. Levels of fluorescence associated with hydrolysis of quenched fluorogenic peptide substrates are measured at 320 nm excitation and 405 nm emission. Images of the scratch wounded cells are acquired every 2 h using an ORCA-R2 Digital CCD camera (C10600, Hamamatsu) mounted on an automated Zeiss Axio Imager Z1 microscope equipped with a 5× objective (N-Achroplan 5×/0.13, Zeiss) and temperature controlled environmental chamber set to 37°C. We analyse images using ImageJ software to determine the percentage of wound closure, or relative invasion, where *w*(*t*) is the area covered by cells at time *t*:
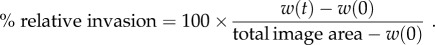


### 2.3. Scanning electron microscopy imaging

Collagen gels are fixed in 2.5% glutaraldehyde solution (0.1 M sodium phosphate buffer, pH 7.2) for 2 h at room temperature, followed by post-fixation in 1% osmium tetroxide (0.1 M sodium phosphate buffer, pH 7.2) for 1 h at room temperature. Samples are then dehydrated using a graded ethanol series (30%, 60%, 80%, 100% ethanol, for 10 min each), critical-point dried (Tousimis), sputter-coated with 1–2 nm of gold (Denton) and imaged by scanning electron microscopy (JEOL).

### 2.4. mRNA/miR isolation and quantitative polymerase chain reaction

Relative gene expression levels are measured using the Applied Biosystems 7300 Real-Time PCR System. Briefly, total RNA is extracted from cells using the miRNeasy Mini Kit (Qiagen) following the manufacturer's protocol. RNA purity and concentration are measured using an ND-100 Nanodrop spectrophotometer (Thermo Scientific). Total RNA is reverse transcribed (High Capacity cDNA Reverse Transcription Kit, Applied Biosystems) using 500 ng of RNA for each 20 μl reaction. We perform real-time polymerase chain reaction (qRT-PCR) using TaqMan Gene Expression Assays (Applied Biosystems) in a final volume of 20 μl containing 4 ng of template. Expression levels are normalized to levels of 18S and calculated as fold change (2^−ΔΔCT^) with respect to the scrambled negative control samples. To obtain absolute quantification of miR levels, we use droplet digital PCR (Bio-Rad). Purified RNA is polyadenylated using poly-A polymerase (New England Biolabs) following the manufacturer's protocol and reverse transcribed using the iScript Select cDNA Synthesis Kit (Bio-Rad). We measure the number of miRs detected in 2 µg of RNA for samples with low levels of miRs and 50 ng of total RNA for the samples with miR overexpression. Each 20 µl reaction containing 1× EvaGreenSupermix (Bio-Rad), 1× gene-specific primers and 4 µl of cDNA is partitioned into droplets using the QX200 droplet generator and then transferred to a 96-well plate for PCR amplification using a thermal cycler. After PCR, droplets are analysed on a QX200 droplet reader to measure the target miR concentrations. We calculate the average miR copy number per cell based on the quantity of RNA extracted from approximately 10^5^ cells from three independent experiments.

### 2.5. Predicted miR targeting

To clarify the molecular basis of the mechanotype changes observed with miR overexpression, we identify predicted tumour-suppressor miR targets that are implicated in regulating the mechanical properties of cells, including genes that encode for both structural and signalling proteins that regulate cytoskeletal structure and dynamics. We score genes as potential miR targets using three sets of binding site predictions: TargetScan (v. 7.1) [27], miRmap (201301e) [28] and miRanda-mirSVR [29]; for mirSVR, we use target site predictions with ‘good’ scores for conserved miRs (miR-130b-3p) and for non-conserved miRs (miR-508-5p, miR-508-3p, miR-509-5p and miR-509-3p). We consider predicted targets that are in the top 50th percentile of scores for each method. Because a 3′-UTR can have more than one predicted binding site for a mature strand of a given miR, we use a percentile based on the sum of scores of all predicted sites for a mature strand on a 3′-UTR. The total percentile scores reflect the expected effect of a miR in reducing a transcript's abundance; reporting percentiles facilitates comparing scores among methods.

### 2.6. Parallel microfiltration

To measure the ability of cells to deform through micrometre-scale pores, we use a PMF device [27] with polycarbonate membrane filters of 10 µm pore size (Millipore). To minimize cell–surface interactions, membranes and loading wells are treated with 1% bovine serum albumin in deionized water for 1 h at 37°C. Transfected cells are trypsinized at 72 h post-transfection, filtered through 35 μm filters to minimize cell clusters and resuspended to a final concentration of 9 × 10^5^ cells ml^−1^ in culture medium prior to loading. We determine the density of the cell suspension using an automated cell counter (TC20, Bio-Rad); these measurements also confirm that more than 88% of cells are single cells (*N* = 2) (electronic supplementary material, figure S3*a*,*b*). To measure cell deformability using PMF, we apply a uniform air pressure of 7 or 14 kPa for 45 s, collect the samples retained in the top wells and calculate *percentage retention* = (*m*_f_/*m*_i_) × 100%, where *m*_i_ is the mass of cell suspension loaded and *m*_f_ is the mass remaining in the top well after filtration. Percentage retention depends on the driving pressure, the cell size relative to the pore size and the intrinsic cell mechanical properties [27–29].

### 2.7. Microfluidic device fabrication and operation

For microfluidic deformation experiments, we use standard soft lithography techniques to fabricate polydimethylsiloxane (PDMS) (Sylgard 184 Silicone Elastomer, Dow Corning) devices. To probe cell deformability, we use a device design as previously described [25]. In brief, a master mould of the device is patterned from photoresist onto a silicon wafer. PDMS is moulded onto the wafer and cured at 65°C for 4 h, then removed, cleaned and covalently bonded to glass coverslips after exposure to oxygen plasma. To minimize surface adhesion, we add 0.1% v/v F-127 surfactant (Invitrogen) to cell suspensions. We use an applied pressure of 28 kPa to drive cells in suspension through the device constrictions. The resulting cell deformations are imaged using a high-speed camera (Phantom Miro EX1, Vision Research) mounted on an inverted light microscope (Zeiss) equipped with a 20× objective (20×/0.40 Ph2 Corr, LD Achroplan, Zeiss). Images are analysed using a custom algorithm in Matlab (Mathworks) to extract cell size, which is determined before each cell enters the constriction, and transit time; stiffer cells tend to have longer transit times [26,29–31].

### 2.8. Imaging cells and subcellular structures

To visualize F-actin and determine cell/nuclear size, we image cells in suspension using the ImageStream multispectral imaging flow cytometer (Amnis Corporation). Cells are fixed in 4% paraformaldehyde and permeabilized with 0.2% Triton X-100. After blocking, cells are incubated with Alexa Fluor 488-phalloidin (Thermo Scientific), followed by DRAQ5 nuclear stain (Thermo Scientific). We measure morphological features such as cell size, nuclear size and cortical-to-intracellular F-actin levels using the ImageStream Data Analysis and Exploration Software (IDEAS) (Amnis Corporation). To assess the distribution of F-actin, we determine the ratio of cortical-to-intracellular F-actin levels, where the cortical F-actin is measured by the average pixel intensity within 0 < 7 pixels (0 < 3.5 µm) from the boundary of the cell, and intracellular F-actin is measured by the average pixel intensity within the internal region that is more than 3.5 µm from the boundary of the cell. Cell and nuclear sizes are determined by measuring the projected area and extracting the diameter assuming a perfect circle. To visualize F-actin in adhered cells, cells are seeded on collagen-coated glass slides, fixed with 4% paraformaldehyde and labelled with Alexa Fluor 488-phalloidin. We perform confocal imaging using a Zeiss laser scanning microscope (LSM 5 Exciter) equipped with a 63× objective (63×/1.2 W Korr UV-VIS-IR, C-APOCHROMAT, Zeiss), and argon ion (488 nm) and helium-neon lasers (633 nm) for excitation. We measure the total integrated fluorescence intensity of F-actin for individual cells using ImageJ and determine the fold-change fluorescence relative to the SCR treatment.

## 3. Results and discussion

In our previous work, we showed that higher expression levels of the tumour-suppressor miRs miR-508-3p, miR-508-5p, miR-509-3p, miR-509-5p and miR-130b-3p in human ovarian tumours correlate with improved patient prognosis and can discriminate improved survival in HGSC patients [3,32]. Here, to obtain fold-change increases in miR levels that are similar to the increased levels between cancer patients at early versus advanced stages of disease [33,34], we transiently transfect miRs into HEYA8 and OVCAR8 ovarian cancer cells.

### 3.1. Quantification of miR expression levels

**Figure 1.**
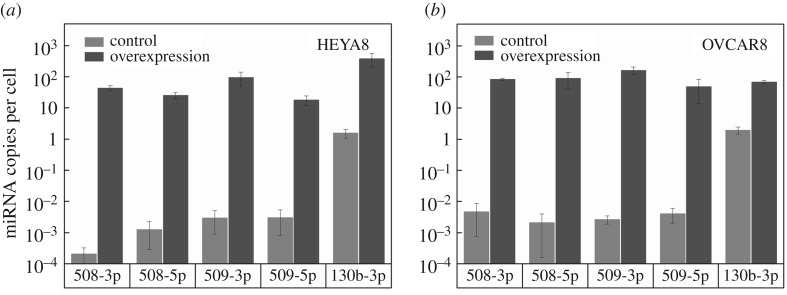
Quantification of miR levels using droplet digital PCR. MiR copy number in negative control and miR-overexpressing samples 72 h after transfection with tumour-suppressor miRs. Data are shown for (*a*) HEYA8 and (*b*) OVCAR8 cells. The average numbers of miR copies per cell are calculated based on the quantity of RNA extracted from approximately 10^5^ cells. Error bars show the standard deviation of *N* = 2 independent experiments.

To determine how the fold-change increase in tumour-suppressor miRs with overexpression relates to clinical contexts, we first measure endogenous levels of miRs in HEYA8 and OVCAR8 cells using droplet digital PCR (figure 1). Our analysis reveals that these tumour-suppressor miRs have endogenously low copy numbers in the non-treated cells. For example, in HEYA8 cells, miR-130b-3p is present at approximately one copy per cell, while miR-508-3p, miR-508-5p, miR-509-3p and miR-509-5p are even less abundant, ranging from 10^−4^ to 10^−3^ copies per cell (figure 1*a*). Overall, the OVCAR8 cells show similar endogenous levels of miRs compared to the HEYA8 cells (figure 1*b*). Following transient transfection of these miRs, expression levels in HEYA8 increase by up to five orders of magnitude up to 20–120 copies per cell 72 h after transfection. The cells treated with miR-508-3p show the largest fold increase of approximately 10^5^, while levels of miR-130b-3p increase by approximately 10^2^-fold. For OVCAR8, we observe up to an approximately 10^4^ fold increase in miR levels, with 50–160 copies per cell 72 h after transfection. Across the panel of tumour-suppressor miRs, we observe on average a 10^4^-fold increase in miR levels. These transient transfections generate changes in the levels of tumour-suppressor miRs that are similar to those observed in clinically relevant contexts. For example, miR-508-3p, miR-509-3p and miR-509-5p are less abundant by approximately 10 to 10^3^-fold in ovarian cancer patients with stage III versus stage I disease [33], and miR-508-3p and miR-508-5p are approximately 10–10^2^-fold less abundant in metastatic serous epithelial ovarian tumours than in primary tumours [34].

### 3.2. MiR overexpression hinders cell invasion through collagen matrices

**Figure 2.**
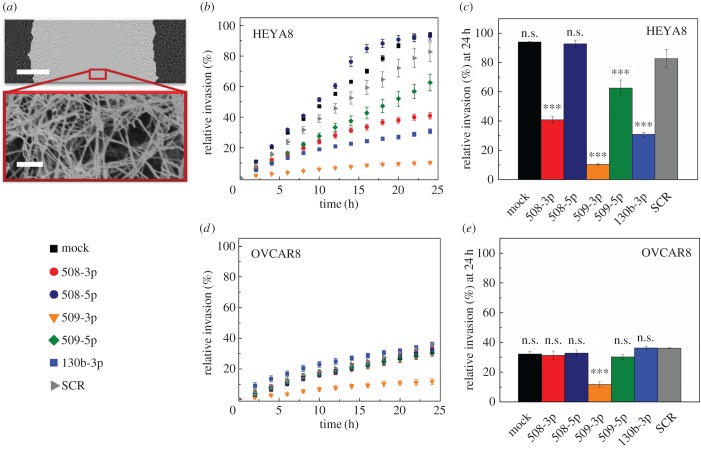
Effect of miR overexpression on cell invasion through a collagen matrix. (*a*) Scratch wound assay where cells invade through a collagen gel. The upper panel shows a top view of the scratch; the cells on right and left sides invade into this wounded region, which is overlaid with a collagen gel. Scale bar, 200 µm. The bottom panel shows a scanning electron micrograph of the fibrous structure of a 1 mg ml^−1^ collagen gel. Scale bar, 1 µm. (*b*,*d*) Invasion time course for HEYA8 (*N* = 3) and OVCAR8 (*N* = 3) cells in 1 mg ml^–1^ collagen type I in the presence of a protease inhibitor, 50 µM GM6001. (*c*,*e*) Relative invasion of HEYA8 and OVCAR8 cells at 24 h for different miR treatments. One-way ANOVA with a Tukey post hoc test: ***p < 0.001, n.s., *p* > 0.05, compared to the scrambled control treatment (SCR). All error bars show the standard error of the mean.

To deform through gaps of the ECM, invading cells undergo major changes in shape that are associated with cytoskeleton remodelling. The magnitude of cell deformation required to pass through narrow pores depends on the ECM network mesh size and mechanical properties of the fibrous proteins. To determine the extent to which elevated levels of miRs hinder cell invasion through an ECM, we perform invasion assays using a type I collagen matrix (figure 2*a*), which is the most abundant structural component of the ovarian ECM [35]. Scanning electron microscopy (SEM) of the 1 mg ml^−1^ collagen gels that we use in our invasion assay reveals that the average gap size is 0.5–2 µm; this is significantly smaller than the 23–25 μm median diameter of cells overexpressing the tumour-suppressor miRs. Invading tumour cells can also increase the matrix gap size by secreting proteases that degrade collagen. Indeed, elevated levels of several MMPs, including MMP-2 and MMP-9, in ovarian cancer cells are associated with increased invasive potential, and with poor survival of ovarian carcinoma patients [36–38]. To reduce the potential role of matrix degradation and thereby enhance the requirement for cells to deform during invasion, we treat cells with the broadband MMP inhibitor GM6001, which suppresses the invasion of cells through type I collagen matrices [39]. We confirm that GM6001 decreases protease activity in conditioned medium taken from cell culture 24 h after treatment with GM6001 (electronic supplementary material, figure S1).

Over the 24-h invasion time course, the HEYA8 mock and SCR-treated control cells show progressive closure of the scratch wound. We observe no significant differences between mock and SCR-treated cells (94%, 83% relative invasion, respectively, *p* = 0.33). By the endpoint of 24 h, the wound is nearly completely healed, with 83–94% wound closure (figure 2*b*,*c*). By contrast, HEYA8 cells overexpressing miR-508-3p, miR-509-3p and miR-130b-3p show a marked reduction in wound closure (41%, 10%, 31% relative invasion, respectively, *p* < 0.001). Cells with elevated levels of miR-509-5p also show significantly reduced invasion compared to the mock and SCR cells (63% relative invasion, *p* < 0.001). Across our miR panel, we observe a significant decrease in wound closure rate with overexpression of all miRs except miR-508-5p. Compared to the HEYA8 cells, OVCAR8 cells show reduced invasion of the mock and SCR controls (34–36% relative invasion, *p* < 0.001). The more rapid invasion of HEYA8 cells compared to OVCAR8 cells is consistent with the mesenchymal phenotype of the HEYA8 cells versus the epithelial-like OVCAR8 cells; cells become more motile during epithelial-to-mesenchymal transition (EMT) [40]. Only miR-509-3p shows a significant decrease in wound closure and speed of the wound front in both cell lines (electronic supplementary material, figure S2; *p* < 0.001 in each cell line). The reduced invasion of miR-509-3p-overexpressing cells through collagen matrices is consistent with previous results showing decreased invasion of ovarian cancer cells through Matrigel [3], as well as reduced migration of human epithelial lung cancer and renal cancer cells in scratch wound assays [5,6]. Our results are also in agreement with other studies that show reduced invasion with overexpression of miR-508-3p in gastric [41] and renal [5] cancers, as well as with overexpression of miR-130b-3p in pancreatic cancer [42].

### 3.3. Elevated levels of miRs alter cell physical properties

**Figure 3.**
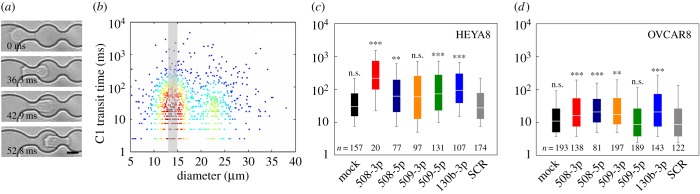
Cells that overexpress tumour-suppressor miRs have longer transit times through microfluidic constrictions. (*a*) We apply air pressure of 28 kPa to drive cells through a PDMS microfluidic device with constrictions that have a width of 7 µm and height of 10 µm; the constriction diameter is approximately half the median cell size of 23–25 µm. Cell deformations are imaged using a high-speed camera and analysed using a custom image processing algorithm to extract the time for each cell to transit through the first constriction (C1). Micrographs show a representative HEYA8 cell that has a transit time of 42.9 ms. Scale bar, 10 µm. (*b*) Density scatter plot showing relationship between cell size and transit time for *N* = 889 individual SCR-treated HEYA8 cells. The shaded grey region indicates cells with an apparent diameter of 13–15 µm; we apply this size filter to compare cells of similar sizes across the miR panel. An upstream filter minimizes larger cells and cell aggregates from entering the constriction region. (*c*,*d*) Distributions of transit times for HEYA8 and OVCAR8 cells treated with tumour-suppressor miRs. Each panel displays the number of cells per sample, which are compiled over *N* = 2 independent experiments. Horizontal lines denote medians, boxes represent the 25th and 75th percentiles and whiskers show the 10th and 90th percentiles. SCR, scrambled control. Mann–Whitney test: n.s., not significant; ***p < 0.001, **p < 0.01, compared to the SCR treatment.

To determine the effect of elevated levels of tumour-suppressor miRs on cell deformability, we perform a microfluidic deformation assay that enables us to measure both the size and transit time of single cells as they deform and pass through a micrometre-scale channel. In this assay, the time for transit exhibits a strong dependence on cell deformability, with cells that are more compliant having shorter transit times than stiffer cells [28,30,31,43,44]. Transit time also shows a weak dependence on cell size [30,45]; however, larger cells get filtered out in the upstream filter region of the device and we further size filter the data to compare the transit times for cells of similar sizes across our miR panel (figure 3*b*). Both HEYA8 and OVCAR8 cells that overexpress miR-508-3p, miR-508-5p and miR-130b-3p have significantly longer transit times compared with the mock and SCR controls (figure 3*c*,*d*). For miR-508-3p, we observe the longest median transit time in HEYA8, and also an increased median transit time for OVCAR8 compared to SCR (approx. 8× longer median transit time for HEYA8, 2× for OVCAR8; *p* < 0.001). For the OVCAR8 cells, we find an approximately 3× increased median transit time with overexpression of miR-130b-3p compared to the SCR cells (*p* < 0.001). We find a similar 2× increase in median transit time for both cell lines with overexpression of miR-509-3p compared to SCR control cells (OVCAR8, *p* = 0.002; HEYA8, *p* = 0.06). Our results also show a 3× increased median transit time with overexpression of miR-509-5p in HEYA8 cells compared to SCR (*p* < 0.001), but no significant change for OVCAR8 cells (*p* = 0.87). Overall, our results show that overexpression of tumour-suppressor miRs results in an increase in transit time, indicating that cells overexpressing these miRs are less deformable.

**Figure 4.**
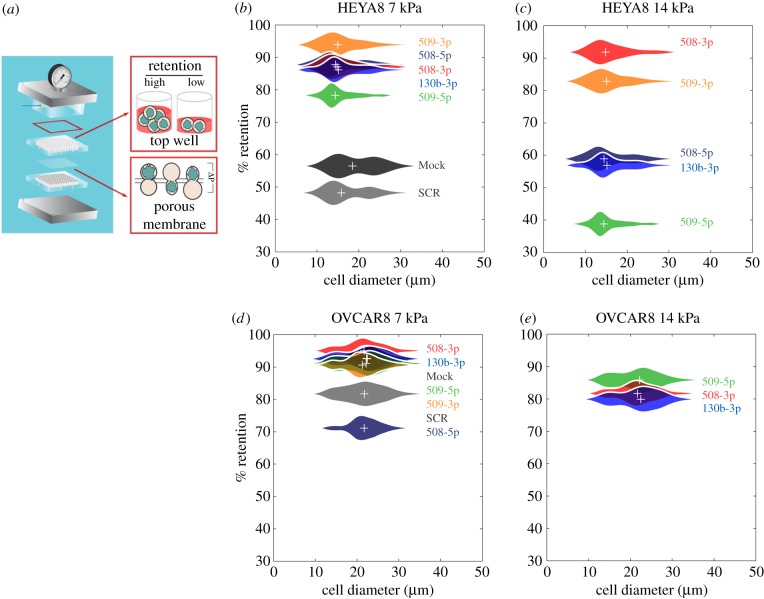
Cells that overexpress tumour-suppressor miRs show decreased filtration through a porous membrane. (*a*) Schematic of the PMF set-up. Figure adapted from Qi *et al*. [27]. A cell suspension is driven to flow through a membrane with 10 µm pores by applying air pressure across the multiwell device; a larger number of occluded pores results in increased retention. Percentage retention as a function of the cell-to-pore size ratio, after 45 s of filtration of (*b*,*c*) HEYA8 and (*d*,*e*) OVCAR8 cells with applied pressures of (left) 7 kPa and (right) 14 kPa. Data are from *N* = 3 independent experiments. Violin plots show cell size distributions; crosses denotes the average retentions and median cell sizes.

To confirm measurements of cell deformability using an independent assay, we use PMF [27]. In PMF, a bulk suspension of cells is filtered through a membrane with 10 µm pores in response to applied air pressure (figure 4*a*). We measure the percentage retention by determining the fraction of sample volume that is retained in the top loading well after applying pressure for a defined period of time. Cells that are less deformable are more likely to occlude the pores, resulting in higher retention; a larger cell size could also increase retention. Given the potential confounding effects of cell size on filtration, we display our retention data together with cell size distributions (figure 4*b*–*e*). We observe that cells overexpressing different miRs exhibit similar cell size distributions, with median sizes that are within ±2 µm (figure 4*b*–*e*; electronic supplementary material, table S1 and figure S5*a*); these results suggest that differences in retention are not strongly influenced by cell size but instead reflect differences in cell deformability among the different miR treatments.

Transfected HEYA8 cells that overexpress tumour-suppressor miRs consistently show a retention that is approximately 30–40% higher than the mock and SCR controls after 45 s of filtration through 10 µm pores at 7 kPa (*p* < 0.001) (figure 4*b*). To resolve differences in retention between cells with different miR treatments, we increase the applied pressure. At a driving pressure of 14 kPa, we observe that the mock and SCR control samples completely filter through the membrane on the experimental timescale; this is consistent with our PMF results at lower pressure, where mock and SCR exhibit the lowest retention, indicating these cells are the most deformable (figure 4*c*). HEYA8 cells that overexpress tumour-suppressor miRs show higher retention at 14 kPa, reflecting that more of these cells occlude the 10 µm pores even at this higher driving pressure. Cells with elevated levels of miR-508-3p and miR-509-3p show the highest retentions (92 ± 5% and 83 ± 5%, respectively) compared to the other miR treatments (*p* < 0.001), while retention of miR-508-5p (59 ± 5%), miR-509-5p (39 ± 5%) and miR-130b-3p (56 ± 5%) overexpressing cells is still significantly higher than the control samples at this increased driving pressure (figure 3*c*) (*p* < 0.001). By contrast, OVCAR8 cells show higher levels of retention than HEYA8 at the same pressures; this is consistent with the more invasive HEYA8 cells being more deformable. For OVCAR8 cells at 14 kPa driving pressure, we observe higher retention for miR-508-3p (82 ± 11%), miR-509-5p (86 ± 6%) and miR-130b-3p (80 ± 8%) compared to the mock and SCR (*p* < 0.001), but no significant change in retention for cells treated with miR-508-5p (*p* = 0.9) and miR-509-3p (*p* = 0.9). Overall, we find that miR-508-3p results in the largest increase in retention in both HEYA8 and OVCAR8 cells. Overexpression of miR-509-5p and miR-130b-3p also results in consistently higher retention relative to the SCR control in both cell types.

The results of these complementary filtration and microfluidic methods to measure cell deformability are in good agreement. Overexpression of miR-508-3p in both HEYA8 and OVCAR8 causes increased retention and transit time, reflecting decreased cell deformability. While both methods probe the ability of cells to transit through micrometre-scale pores, we do not observe the same trends for all miRs; this could reflect differences in the readouts for the bulk (PMF) versus single cell (transit time) deformability assays (see the electronic supplementary material for further discussion). For example, overexpression of miR-509-3p in HEYA8 cells results in a slight shift to increased transit times, but a statistically significant increase in retention compared with SCR cells; this could result from occlusion due to the longer transiting cells.

### 3.4. Prioritizing genes of the mechanome that are implicated in cytoskeletal structure and dynamics

**Figure 5.**
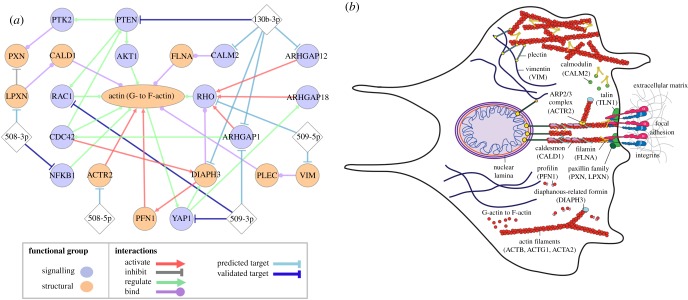
Predicted effects of tumour-suppressor miRs on cytoskeletal structure and dynamics. (*a*) Network showing predicted and validated miR targets and functionally annotated protein–protein interactions for structural and signalling proteins that are known to regulate the mechanical properties of cells. Predicted miR–mRNA targets are determined using TargetScan v. 7.1, miRanda-mirSVR and miRmap; all targets displayed have scores in the top 50th percentile of predicted targets for all three methods, except for RAC1, which is a validated target of miR-509-3p [6]. Experimentally validated direct miR–mRNA targets are denoted by dark blue lines. Diamonds represent miRs; circles represent genes; and the ellipse represents actin, which exists as both monomers (G-actin) and filaments (F-actin). RHO denotes RhoA, RhoB and RhoC, which have high sequence homology [47–49]. While RHOC is a highly scoring predicted target of miR-509-5p, and RHOA is regulated by ARHGAP18 [50], the preferred substrates for other RhoGTPase-activating proteins are not fully understood. (*b*) Schematic illustrating regulation of the architecture and dynamics of the actin cytoskeleton.

To determine the mechanisms through which the miRs in our panel could be inducing changes in cell physical properties, and ultimately invasive behaviour, we determine predicted targets of miRs that could be implicated in cell mechanical properties. We prioritize genes that encode major cytoskeletal proteins (such as vimentin), proteins that interact with components of the cytoskeleton (actin cross-linkers like filamin A), as well as proteins that regulate cytoskeletal organization (NF-κB). We assess the probability of miR-gene targeting that influences mRNA transcript levels using three independent and favourably ranked [46] sets of binding site predictions: TargetScan (v. 7.1) [27], miRmap (201301e) [28] and miRanda-mirSVR [29]. To enable comparisons of the predictions from each resource, we assess the binding site scores as percentiles with values between 0 and 1, and report only genes that are in the top 50th percentile of targets for all three resources, or are validated targets (figure 5*a*). Below, we summarize predicted and validated targeting that is relevant for the effects of our tumour-suppressor miRs on cell deformability.

#### 3.4.1. miR-508-3p

**NFKB1**, which encodes the protein NF-κB, is a predicted target of miR-508-3p (TargetScan = 0.96, mirSVR = 0.90, miRmap = 0.82). This miR is validated as inactivating canonical NF-κB signalling in gastric cancer cell lines and primary tumours [41,51,52]. NF-κB disrupts tissue organization and cellular morphology; it is a regulator of genes involved in actin organization and cell adhesion in breast cancer cells [53]. Another predicted target of miR-508-3p is leupaxin (**LPXN**) (TargetScan = 0.97, mirSVR = 0.73, miRmap = 0.62), which is a member of the paxillin family of focal adhesion proteins. Leupaxin directly affects cytoskeletal organization and dynamics through its interaction with the actin-binding protein caldesmon (**CALD1**) [54,55].

#### 3.4.2. miR-508-5p

**ACTR2** is a highly scoring predicted target of miR-508-3p (TargetScan = 0.99, mirSVR = 0.94, miRmap = 0.89), which encodes a major constituent of the ARP2/3 complex that is essential for lamellipodial actin assembly and protrusion formation, both of which are implicated in cell motility [56].

#### 3.4.3. miR-509-3p

Predicted targets of this miR include **YAP1** (TargetScan = 0.96, mirSVR = 0.95, miRmap = 0.97), which encodes the Yes associated transcription factor Yap1 that is implicated in regulation of tissue tension, as well as in cell shape and migration [57]. The effects of Yap1 on the actin cytoskeleton are regulated via the Rho GTPase, **ARHGAP18**, which suppresses F-actin polymerization by inhibiting RhoC [58] and RhoA [50]. Recently, we validated YAP1 as a direct target of miR-509-3p [3]. We also identify **ARHGAP1**, the Rho GTPase-activating protein 1, as a potential target of miR-509-3p (TargetScan = 0.92, mirSVR = 0.76, miRmap = 0.78); this protein also regulates assembly of F-actin [59]. While **RAC1**, which is a member of the Rho family of GTPases, scores highly as a predicted target using only one method (TargetScan = 0.99), it is a validated target of miR-509-3p [6]. Rac1 contributes to the formation of membrane protrusions and cell–matrix adhesions that are essential in cell motility [60] and is also overexpressed in several types of tumours [61–63].

#### 3.4.4. miR-509-5p

The intermediate filament protein, vimentin (**VIM**), is a highly scoring predicted target of miR-509-5p (TargetScan = 0.97, mirSVR = 1.00, miRmap = 0.95). Vimentin is widely used as a biomarker for mesenchymal-type cells. This protein is also observed to promote cell motility and increase focal adhesion dynamics during epithelial-to-mesenchymal transition [64]. Depletion of vimentin also results in reduced cell stiffness [65–67]. MiR-508-5p is also predicted to target **RHOC** (TargetScan = 0.99, mirSVR = 0.66, miRmap = 0.77), which encodes a Rho GTPase protein that plays an important role in cell motility [48,49]. RhoC expression positively correlates with cancer metastasis in melanoma and breast cancer [49,68,69].

#### 3.4.5. miR-130b-3p

The tumour-suppressor **PTEN**, encoding phosphatidylinositol-3,4,5-trisphosphate 3-phosphatase, is a validated target of miR-130b-3p [70] that is also highly scored as a predicted target (TargetScan = 0.94, mirSVR = 0.87, miRmap = 0.95). Downregulation of PTEN promotes the motility of fibroblasts through stimulation of Cdc42 and Rac1 activity [70–72]. Another predicted target of miR-130b-3p is **DIAPH3** (TargetScan = 0.67, mirSVR = 0.81, miRmap = 0.93), which encodes diaphanous-related formin-3 (Drf3). Drf3 binds to profilin (**PFN1**) [73,74] and guides its downstream effector, Cdc42, to the cell cortex where it plays a role in remodelling the actin cytoskeleton [75]. This formin is essential in multiple processes that rely on actin polymerization, such as blebbing [76] and actin nucleation in the formation of cellular protrusions [77]; it also localizes to stress fibres and filopodia [78]. MiR-130b-3p is also predicted to target **CALM2**, the calcium-binding protein calmodulin (TargetScan = 0.99, mirSVR = 0.85, miRmap = 0.83). Complexes of calmodulin and Ca^2+^ bind to the actin-binding domain of filamin A, an actin cross-linking protein, dissociating filamin A from F-actin, thereby dissolving gels of filamin A and F-actin [79]. The Rho GTPase-activating proteins 1 (**ARHGAP1**) and 12 (**ARHGAP12**) are also predicted targets of miR-130b-3p (ARHGAP1: TargetScan = 0.94, mirSVR = 0.76, miRmap = 0.90; ARHGAP12: TargetScan = 0.97, mirSVR = 0.79, miRmap = 0.95). Rho GTPase-activating proteins (GAPs) stimulate Rho GTPase activity and promote the conversion of active GTP-bound proteins to the inactive GDP-bound state. In the activated state, Rho GTPases regulate F-actin assembly and enhance stress fibre formation [80,81].

### 3.5. Expression of key mechanoregulating genes is altered by tumour-suppressor miRs

To experimentally confirm the altered expression of select candidate targets following miR overexpression, we measure transcript levels at 72 h post-transfection using real-time polymerase chain reaction (qRT-PCR). We observe a significant increase in ACTA2 (smooth muscle α-actin; two- to sixfold) following treatment with miR-508-3p, miR-508-5p and miR-130b-3p in HEYA8 cells (*p* < 0.01), but there is no observable change following miR-509-3p overexpression (*p* > 0.05). Overexpression of miR-130b-3p also reduces invasion and shows an approximate twofold increase of ACTA2 in HEYA8 cells (*p* < 0.01). While miR-508-5p also results in a similar approximately twofold higher expression of ACTA2 in HEYA8 cells (*p* < 0.01) and a small increase in transit time and retention (*p* < 0.01), we observe no significant effect on invasion (*p* = 0.4). For OVCAR8 cells, only miR-130b-3p results in a significant change in ACTA2 levels (*p* < 0.001); in contrast to HEYA8 cells, there is a decrease in ACTA2 levels in OVCAR8 cells (*p* < 0.001). Smooth muscle α-actin associates predominantly with stress fibres or microfilament bundles [82], and its expression correlates with impaired motility in fibroblasts [83]. However, increased ACTA2 levels are also associated with invasion in breast and lung cancer [84,85]. The role of β-actin isoforms, which are encoded by ACTB, in cytoskeletal organization and cell migration is less well understood; β-actin is observed to accumulate in cellular protrusions involved in motility [86], while other studies report that β-actin is predominantly found in stress fibres and at cell–cell adhesions [87]. ACTB levels are not significantly altered in HEYA8 cells, but we observe a significant decrease with overexpression of miR-508-3p (*p* < 0.01), miR-509-3p (*p* < 0.001) and miR-509-5p (*p* < 0.05) in OVCAR8 cells.

In addition to actin, we identify VIM and YAP1 as predicted miR targets and regulators of cell mechanical properties and mechanotransduction. With overexpression of miR-509-5p, we observe decreased vimentin expression in both HEYA8 (*p* = 0.07) and OVCAR8 cells (*p* < 0.001); these observations are consistent with our target predictions (VIM: TargetScan = 0.97, mirSVR = 1.00, miRmap = 0.95; figure 5). We also observe changes in vimentin levels with overexpression of other tumour-suppressor miRs. For example, in HEYA8 cells we observe an approximate twofold (*p* = 0.003) increase in transcripts encoding for vimentin with miR-508-3p; by contrast, OVCAR8 cells exhibit a less than twofold decrease in vimentin with miR-508-3p (*p* = 0.04), miR-509-3p (*p* = 0.003) and miR-130b-3p (*p* = 0.03). Increased expression of vimentin is a marker for EMT, which is associated with disease progression [64]; however, the role of vimentin in cancer cell mechanical properties is not fully understood [66,88]. As a cytoskeletal intermediate filament protein, vimentin can contribute to cell stiffness [65–67] and may enable cells to resist large deformations: *in vitro* vimentin networks stiffen when deformed by large stresses that can rupture actin networks [89]. Vimentin also binds to plectin which mediates interactions of intermediate filaments with actin and microtubules [90–92] (figure 5*b*).

**Figure 6.**
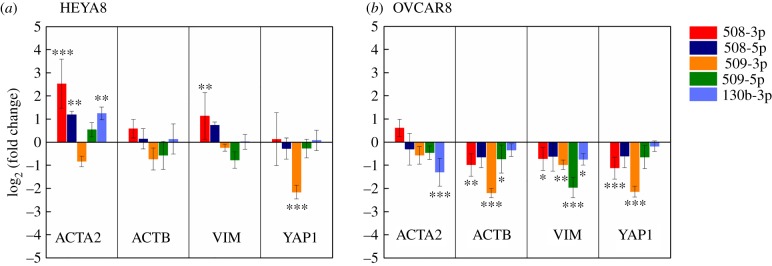
Expression of mechanoregulating genes is altered by overexpression of tumour-suppressor miRs. Quantification of gene expression levels in miR-transfected (*a*) HEYA8 and (*b*) OVCAR8 cells relative to the SCR negative control, as measured by qRT-PCR using the delta delta cycle time method (ΔΔCt) with 18S ribosomal RNA as an endogenous control. Error bars show standard deviations of *N* = 3 independent experiments. One-way ANOVA with a Tukey post hoc test: ***p < 0.001, **p < 0.01, *p < 0.05, compared to the scrambled control treatment (SCR).

YAP1 is a predicted target of miR-509-3p (TargetScan = 0.96, mirSVR = 0.95, miRmap = 0.97), and a transcriptional co-activator and oncogene that is implicated in sensing mechanical cues and intracellular tension [93,94]; Yap1 localization is sensitive to cell shape and polarity [93,95–97]. With miR-509-3p overexpression, we observe a significant approximately fivefold downregulation of YAP1 in both HEYA8 and OVCAR8 cells (*p* < 0.001) (figure 6). We previously established YAP1 to be a direct downstream target of miR-509-3p [3]. There is also an approximate twofold downregulation of YAP1 with miR-508-3p treatment in OVCAR8 cells (*p* = 0.003) but no significant change in HEYA8 cells. Since Yap1 is implicated in sensing cytoskeletal tension [93,94], we speculate that this protein could influence invasive behaviour by dynamically regulating cell deformability and traction forces as a cell migrates through narrow gaps. However, the precise role of YAP1 in regulating the invasive potential and deformability of single cells remains to be clarified.

### 3.6. Understanding the origins of altered deformability and invasive behaviour

To further dissect the origins of the increased retention and transit times that we observe with transfection of tumour-suppressor miRs, we identify genes associated with regulation of the cytoskeleton, which is a key regulator of cell mechanotype [20,98]. The network shown in figure 5*a* focuses on miRNA–mRNA and protein–protein interactions; complementing this, the relevant molecular complexes and structures that can determine cell physical properties are illustrated in the schematic shown in figure 5*b*. Many of the predicted and validated targets converge on regulation of the actin cytoskeleton. Actin is a major cytoskeletal protein that has three main isoforms in mammalian cells: α-actins are a major component of the contractile apparatus, while β and γ-actins primarily regulate cell motility. These actin isoforms exist as monomers (G-actin), which can polymerize to form filaments (F-actin) that play a key role in the morphological changes and physical forces generated during migration [99,100]. The role of actin in regulating single-cell mechanical properties is well established [20,31,44]. Here, we experimentally confirm the predicted changes in actin levels in the HEYA8 cells (figure 5*a*), which show a more pronounced increase in monomeric actin levels than in the OVCAR8 cells with miR overexpression.

**Figure 7.**
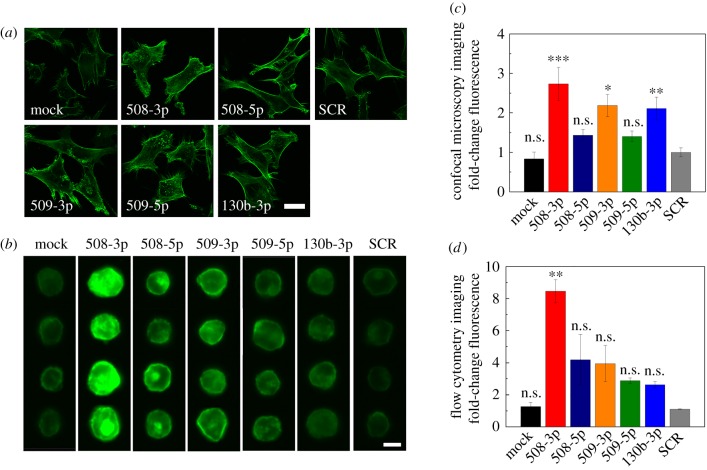
Subcellular structure with elevated levels of tumour-suppressor miRs. (*a*) Confocal microscopy images of HEYA8 cells attached to collagen-coated glass slides. Scale bar, 20 µm. (*b*) Representative images of HEYA8 cells in suspension obtained by flow cytometry imaging. Scale bar, 10 µm. (*c*) Measurements of the integrated fluorescence intensity of single cells in the adhered state. At least 24 cells are analysed for each sample over *N* = 3 independent experiments. (*d*) Quantification of the relative fluorescence intensity of F-actin per single cell based on flow cytometry imaging. At least 3991 cells are analysed for each sample over *N* = 3 independent experiments. One-way ANOVA with a Tukey post hoc test; ***p < 0.001, **p < 0.01, n.s., *p* > 0.05, compared to the scrambled control treatment (SCR). Error bars show standard error of the mean.

To quantify levels and organization of F-actin in populations of miR-treated cells, we investigate cells that are adhered to a collagen-coated substrate and labelled with phalloidin; cells also adhere to collagen during invasion (figure 2) [12]. We find that HEYA8 cells with elevated levels of miR-508-3p, miR-509-3p and miR-130b-3p show a two- to threefold increase in F-actin levels (*p* < 0.05), whereas cells with elevated levels of miR-508-5p and miR-509-5p show a slight, albeit not statistically significant, increase in F-actin (*p* = 0.83 and 0.84, respectively; figure 7*c*).

While knowledge of actin structure in adhered cells is relevant for invasion, investigating F-actin in suspended cells is relevant to our deformability assays, which measure cells in suspension. Knowledge of subcellular architecture for suspended cells may also be relevant because cells transit and deform through vasculature during metastasis [101]. We next use imaging flow cytometry to image single cells in suspension, after being fixed and stained with phalloidin. Representative images are shown in figure 7*b*. Compared to the mock and SCR controls, cells overexpressing tumour-suppressor miRs have increased F-actin levels (figure 7*b*,*d*). We observe the largest increase in F-actin levels for cells that overexpress miR-508-3p, which exhibit approximately eightfold higher levels of relative fluorescence intensity compared to the mock and SCR controls (*p* = 0.003; figure 7*d*). Cells overexpressing other tumour-suppressor miRs (miR-508-5p, miR-509-3p, miR-509-5p and miR-130b-3p) show approximately two- to fourfold higher levels of F-actin relative to the controls (*p* > 0.2). These increased levels of F-actin are consistent with the longer transit times for cells overexpressing tumour-suppressor miRs (*R* = 0.92, *p* = 0.003) and reflect how cytoskeletal organization determines transit time and cell deformability.

We also investigate levels of F-actin in the cortical region, as these are critical for the deformability of cells in suspension [102,103]. Interestingly, our results show that cells with elevated levels of miR-508-3p, miR-509-3p or miR-130b-3p, which are less deformable, have lower levels of cortical-to-internal F-actin than the mock and SCR cells (*p* < 0.001; electronic supplementary material, figure S6). While lower levels of cortical F-actin are typically associated with a more deformable cortical region, as measured by methods that induce nanometre to submicrometre deformations [104], our PMF and transit time measurements probe cells as they transit through pores that are roughly half their size. The relatively large deformations in our mechanotyping methods may explain why levels of cortical-to-internal F-actin do not correlate with our deformability results; these observations are in agreement with our previous report that the ratio of cortical-to-internal F-actin cannot predict retention of ovarian cancer cells during EMT [27].

Across the miR panel, we observe a similar trend in overall F-actin levels between cells in an adhered versus suspended state, with smaller fold changes in F-actin levels in adhered cells (figure 7). For example, for cells overexpressing miR-508-3p, there is an eightfold increase in F-actin in suspended cells (*p* = 0.003), and only a threefold increase in adhered cells (*p* < 0.001). The difference in phalloidin intensity between cells in suspended versus adhered states is consistent with how we image them: for flow cytometry imaging, we capture light that is emitted from the entire cell volume, whereas for confocal microscopy, we measure fluorescence signal from a single confocal slice. Taken together, our imaging data for both adhered and suspended cells are generally consistent with our qRT-PCR analyses, which show a significant increase in ACTA2 (1.5 to 6-fold) with overexpression of miR-508-3p, miR-508-5p, miR-509-5p and miR-130b-3p.

**Figure 8.**
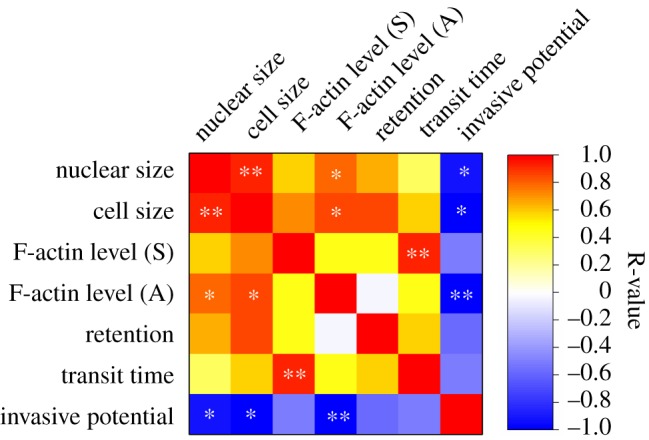
Correlations between invasion, cell physical properties and F-actin levels across the panel of tumour-suppressor miRs. Pearson correlation coefficient (*R*-value) between nuclear size, cell size, F-actin levels in suspended (S) and adhered (A) cells, retention, transit time and invasion of cells with elevated miR expression. *p < 0.05, **p < 0.01 indicate statistically significant correlations.

As changes in the actin cytoskeleton can induce changes in cell volume [105,106], and larger cells exhibit reduced invasion [16,107], we also investigate cell size. To determine differences in cell volume with miR overexpression, we analyse our flow cytometry imaging data; the size of cells in a suspended state can be measured independently from the effects of cell spreading that occur when cells are adhered to a substrate [108,109]. We observe that cells with elevated levels of tumour-suppressor miRs are on average 5–20% larger than the parental cells and negative control-treated cells: the median diameter of cells treated with tumour-suppressor miRs range from 23 to 25 µm, while the SCR control cells have a median diameter of 22 µm (electronic supplementary material, table S1*a* and figure S5*a*). By staining cells with DRAQ5, we also obtain measurements of nuclear size. We observe that the median diameters of nuclei in miR-treated cells range from approximately 11 to 13 µm; this is approximately 10–26% larger than the nuclei of the SCR control cells, which have a median diameter of 10 µm (*p* < 0.001; electronic supplementary material, table S1*b* and figure S5*b*). An increase in cell and nuclear size could contribute to increased transit time and retention; however, cell and nuclear size do not exhibit significant correlations with either transit time (cell size: *R* = 0.61, *p* = 0.14; nuclear size: *R* = 0.36, *p* = 0.42) or retention (cell size: *R* = 0.82, *p* = 0.09; nuclear size: *R* = 0.66, *p* = 0.23; figure 8), suggesting that these deformability measurements are not dominated by cell or nuclear size.

### 3.7. Determining correlations between cell physical properties and invasive behaviour

To summarize the major contributions of cell physical properties to invasive behaviour across the panel of tumour-suppressor miRs, we determine the strength and significance of correlations between invasion and cell size, nuclear size, deformability (retention and transit time) and F-actin levels (figure 8).

#### 3.7.1. Cell invasion is negatively correlated with cell and nuclear size

We find that across the panel of tumour-suppressor miRs, cell size negatively correlates with invasion efficiency (*R* = −0.85, *p* < 0.05); these results are consistent with previous observations in fibroblast (HT-1080), lung (A125) and breast cancer cells (MDA-MB-231) [16,107]. The nucleus is typically the largest and stiffest organelle, occupying a substantial volume of the cytoplasm and rate-limiting the transit of cells through micrometre-scale pores [110–112]; nuclear size is also associated with how effectively cells invade through collagen gels [16]. We observe a moderate negative correlation between invasion and nuclear size (*R* = −0.83, *p* = 0.02), reflecting how overexpression of some miRs results in increased nuclear size as well as reduced invasion, while overexpression of other miRs results in larger nuclei but no significant change in invasion efficiency. Thus, while we observe correlations between invasive potential and cell/nuclear size, this feature alone cannot predict how an individual miR will impact cell invasive potential. Given recent observations of nuclear rupture and repair during the migration of cells through confined geometries [113], it remains to be determined how nuclear size may impact disease progression *in vivo*.

#### 3.7.2. Invasive behaviour of cells is weakly correlated with deformability

To determine the deformability of populations of single cells treated with a panel of miRs, we perform transit time measurements. We also obtain complementary data using PMF, which provides a bulk measure of the deformability of a cell suspension. Considering the panel of miRs, we observe a weak correlation between invasive behaviour and retention (*R* = −0.51, *p* = 0.38) as well as transit time (*R* = −0.43, *p* = 0.33), reflecting that less deformable cells tend to be less invasive. This weak correlation reflects the marked differences in the extent to which tumour-suppressor miRs impact cell deformability and invasive behaviour. We find a statistically significant reduction in both cell deformability and invasive potential for miR-508-3p and miR-509-3p; these observations are consistent with previous results showing that less deformable ovarian and breast cancer cells have decreased invasion efficiency [16,23,24]. However, for other miRs there is little correspondence between deformability and invasion. For example, cells overexpressing miR-508-5p show increased retention but no significant change in their invasive behaviour; cells with elevated levels of miR-130b-3p are significantly less invasive but only moderately less deformable. We note that invasion is an active process during which the cell adheres to a substrate and remodels its cytoskeleton on timescales of minutes to hours, while our deformability measurements probe the passive deformability of cells in suspension on timescales of milliseconds to seconds [114].

#### 3.7.3. F-actin levels for adhered cells show the strongest correlation with invasive potential

Our correlation analyses reveal a moderate negative correlation between wound closure and F-actin levels in adhered cells (*R* = −0.89, *p* = 0.008), suggesting that F-actin could be an indicator of cell invasive potential. For example, in HEYA8 cells miR-508-5p has no significant effect on cell invasive potential (*p* = 0.37), nor on F-actin levels in adhered cells (*p* = 0.82); by contrast, miR-508-3p, miR-509-3p and miR-130b-3p all induce a statistically significant decrease in cell invasion (*p* < 0.001) and increase in F-actin levels (*p* < 0.015). While increased F-actin content is correlated with increased invasion and cell motility in lymphoma cells [115], other studies report that malignant cells exhibit a marked decrease in F-actin compared with normal keratinocytes [116]. Additional factors can also regulate actin structure and organization, including regulatory proteins, which are involved in the potential interaction pathways that we identify, such as DIAPH3 [117]; this formin promotes the formation of actin-rich protrusions involved in cell migration and invasion. For suspended cells, we find a negative albeit not statistically significant correlation between F-actin levels and wound closure (*R* = −0.44, *p* = 0.33), indicating that the organization of cytoskeletal actin is more tightly associated with invasive potential in adhered cells than suspended cells.

### 3.8. Potential mechanisms for how miRs alter cell mechanotype and invasive behaviour

Here, we use predicted and validated targeting relationships to associate the miRs with mRNA targets that encode structural and signalling proteins that regulate cell mechanical properties and mechanosensing. We are thereby able to identify mechanisms by which altered levels of tumour-suppressor miRs may alter regulation of cytoskeletal actin to make cancer cells less deformable and less invasive. Changes in cell mechanotype can occur due to regulation of F-actin structure and dynamics; this can be mediated by actin-associated proteins, such as cross-linkers of actin filaments [118]. Remodelling of the actin cytoskeleton is required for numerous processes in cancer invasion, including invadopodia formation and motility [119,120]. Our work identifies several targets whose potential role in ovarian cancer progression warrants further investigation.

We focus first on dissecting the possible mechanism of the phenotype induced by miR-508-3p overexpression, which includes a strong decrease in invasion, increased transit time and retention. Overexpression of miR-508-3p also results in the largest increase in F-actin and ACTA2 transcript levels in HEYA8 cells. A strong predicted target of miR-508-3p is leupaxin (LPXN). Leupaxin interacts with integrins and regulates the lifetime of adhesion sites [121]; since actin polymerization is required for focal adhesion formation, we speculate that reduced LPXN expression may alter actin remodelling, and thereby affect cell deformability. Leupaxin is also an interaction partner of the actin-binding protein caldesmon, and thereby directly affects cytoskeletal structure and dynamics [54,55]. However, analysis of LPXN expression using TCGA data through cBioPortal reveals upregulation in only 4% of patients [122,123] (electronic supplementary material, figure S7), and may therefore have limited clinical impact. Similarly, miR-508-3p overexpression in the p53-mutant OVCAR8 cells shows no significant effects on invasion.

We find that miR-509-3p overexpression results in significantly reduced invasion for both HEYA8 and OVCAR8, as well as increased transit times and retention, which reflect a trend to decreased deformability. Our results showing reduced YAP1 levels in HEYA8 and OVCAR8 with miR-509-3p overexpression are consistent with Yap1 being a validated target of miR-509-3p in ovarian cancer cells [3]. Downregulation of YAP1 by miR-509-3p could also alter cell mechanical properties by regulation of ARHGAP18; this Rho GTPase-activating protein (RhoGAP) regulates the activity of RhoC [58] and RhoA [50]. Suppression of ARHGAP18 could thus lead to accumulation of active Rho and increased F-actin polymerization, which is consistent with our imaging results for both adhered and suspended cells following overexpression of miR-509-3p. Furthermore, increased levels of miR-509-3p and decreased Yap1 levels are associated with improved overall survival, as determined by analysis of TCGA data [3].

In addition to ARHGAP18, our results reveal that ARHGAP1 and ARHGAP12 are regulators of multiple pathways. ARHGAP1 is another predicted target of miR-509-3p; ARHGAP1 and ARHGAP12 are predicted to bind to miR-130b-3p. Depletion of ARHGAPs could lead to increased activation of Rho proteins and the formation of stress fibres [50,124]; this is consistent with our results showing increased F-actin and decreased cell deformability in HEYA8 cells that overexpress miR-509-3p (figures 3, 4 and 7). Although many RhoGAPs appear to have anti-tumourigenic effects in other cancer types [125–127], ARHGAP1, ARHGAP12 and ARHGAP18 may have clinical relevance in ovarian cancer: analysis of TCGA dataset through cBioPortal [122,123] reveals that across 603 patient tumour samples, there is upregulation or amplification of ARHGAP12 in 11% of patients, of ARHGAP1 in 5% of patients and of ARHGAP18 in 4% of patients (electronic supplementary material, figure S7). These findings warrant further investigations of the role of these ARHGAPs in ovarian cancer cell invasion and disease progression.

Rho proteins are also found in other pathways of our interaction network. For example, RhoC is a predicted target of miR-509-5p. Depletion of this RhoGTPase reduces invasion in different cancer types from prostate to breast cancer [128]. RhoC is also a validated target of miR-493 and miR-138, which reduce the migration of cancer cells [129,130]. RhoA is an effector of diaphanous-related formin-3 or DIAPH3, which is implicated in multiple interaction pathways (figure 5*a*). DIAPH3 is in the miR-509-3p/Yap1/DIAPH3/actin axis and is also a predicted target of miR-130b-3p, although overexpression of this miR results in decreased invasion in HEYA8 cells only. While the role of DIAPH3 in cell mechanical properties is poorly understood, DIAPH3 may affect cell deformability through its interactions with actin [78] and microtubules [131]: DIAPH3 remodels the cytoskeleton by nucleating and elongating non-branched actin filaments, thereby promoting F-actin formation [132]. Indeed, the downregulation of DIAPH3 in early to late stage ovarian cancer cells is accompanied by disruption in F-actin accumulation [133]. DIAPH3 also nucleates actin via Cdc42 [75], and thereby contributes to remodelling of the actin cytoskeleton, including the formation of protrusions that are implicated in cell motility. For example, DIAPH3 is enriched in the cellular protrusions of collagen-embedded OVCA429 spheroids, and also contributes to the invasion of ovarian cancer cells in two-dimensional cultures [134]. Consistent with those findings, we find that overexpression of miR-130b-3p, which is predicted to target DIAPH3, results in reduced invasion of HEYA8 cells. DIAPH3 could also impact cell invasive behaviour through the miR-509-3p/Yap1/DIAPH3/actin axis. As expression of DIAPH3 correlates with levels of active Yap1 [135], and overexpression of miR-509-3p reduces Yap1 levels [3], we hypothesize that miR-509-3p overexpression could also decrease DIAPH3 levels, which could destabilize actomyosin structures and thereby cause cells to be less contractile and less invasive.

#### 3.8.1. Differences between HEYA8 and OVCAR8

While we observe that miR-509-3p overexpression has a significant effect on the invasion and deformability of both HEYA8 and OVCAR8 cells, we also observe differences in the behaviours of these two cell lines. For example, we find that miR-508-3p, miR-509-3p and miR-130b-3p all significantly reduce invasion of HEYA8 cells, but do not have a significant effect on the invasive behaviour of OVCAR8 cells. The different trends that we observe between these two cell lines probably reflect their distinct genetic backgrounds: while HEYA8 is p53-wild-type, OVCAR8 is p53-mutant. Mutation of the tumour-suppressor protein p53 may impact levels of key genes in our interaction network, such as ACTA2 and NFKB1. Wild-type p53, but not mutant p53, promotes increased activity of the smooth α-actin promoter [82,136], which is consistent with the higher endogenous levels of ACTA2 in HEYA8 cells versus OVCAR8 that we observe by qRT-PCR (electronic supplementary material, figure S8*a*) and also by analysis of mRNA expression data from The Broad-Novartis Cancer Cell Line Encyclopedia (CCLE) [137] (electronic supplementary material, figure S8*b*). We speculate that the higher endogenous expression of ACTA2 in HEYA8 compared with OVCAR8 cells could lead to the larger increase in ACTA2 levels with miR-508-3p overexpression. Compared to HEYA8 cells, OVCAR8 cells also have lower levels of NFKB1 and higher levels of the Rho GTPase RHOB (electronic supplementary material, figure S8*b*); this is consistent with the suppression of NF-κB by RhoB [138]. As NFKB1 is a highly scoring predicted target of miR-508-3p, overexpression of this miR may have a more significant effect on the invasion behaviour and physical properties of HEYA8 rather than OVCAR8 cells. We also observe that certain predicted miR targets are expressed at different levels in HEYA8 and OVCAR8 cells. For example, a predicted target of miR-130b-3p, ARHGAP12, has slightly lower levels in OVCAR8 than HEYA8 cells (electronic supplementary material, figure S8*a*); therefore, a reduction of ARHGAP12 levels by miR-130b-3p may result in more significant reduction of invasion in HEYA8 cells. In addition to the p53 mutation, OVCAR8 cells have a number of additional mutations compared to HEYA8 cells, which could also result in phenotypic differences between the two cell lines [139]. Differences between HEYA8 and OVCAR8 with miR overexpression may also be related to the 10× greater fold increase in levels of miR-508-3p and miR-130b-3p in HEYA8 than in OVCAR8 cells. Both miR-508-3p and miR-130b-3p exhibit more significant effects on the invasion and mechanotype of HEYA8 cells than of OVCAR8 cells. However, we find there are no significant correlations between miR expression levels, invasive behaviour and physical phenotypes including deformability, F-actin levels and cell/nuclear size (*p* > 0.05); these findings indicate that altered expression of specific miR targets and/or downstream effectors may have a stronger effect than levels of miRs alone. Further studies can provide deeper insight into the effects of tumour-suppressor miRs on the behaviour of different ovarian cancer cell types.

## 4. Conclusion

MiRs play central roles in regulating gene expression and cellular functions across a wide range of biological processes, from embryogenesis to stem cell differentiation to cancer. Here, we dissect the role of single-cell physical properties in cell invasive behaviour following upregulation of tumour-suppressor miRs in two human ovarian cancer cell lines. We focus on a panel of five tumour-suppressor miRs that are expressed at lower levels in later stages of human ovarian cancer and are more abundant in patients with improved survival [3,33,140,141]. The mechanome targets of these miRs that we have identified may be implicated in ovarian cancer progression and warrant further investigation.

Our results also deepen our understanding of how cell/nuclear size, deformability and architecture of the actin cytoskeleton could have functional consequences in cancer invasion. For adhered cells, we find that cell invasive potential is strongly correlated with cellular and nuclear size, as well as F-actin levels. With overexpression of certain miRs, such as miR-508-3p in HEYA8 cells, our results show that cells become less deformable and also less invasive. Thus, our findings also suggest that measurements of cell physical properties could complement existing methods to identify potential therapeutics.

Investigating other physical factors that are implicated in invasion may provide additional insights. Invasion is a complex and dynamic process in which cells remodel the ECM by proteolytic degradation and by exerting traction forces on surrounding ECM fibres. Differences in the adhesive properties of cells could also contribute to the differences in cell invasion that we observe in cells overexpressing tumour-suppressor miRs. In addition to changes in cell physical properties, the ECM also regulates invasion through its mechanical properties and density of cell adhesion binding sites [17,142–144]. Complementary analyses of cell and ECM physical parameters and/or combinations of parameters could thus provide stronger predictive power for diagnosis and treatment response. Ultimately, knowledge of physical properties of tumour cells and their microenvironment could lead to new strategies to better target invasive cancer cells and to reduce metastatic spread.

## Supplementary Material

Electronic Supplementary Material

## References

[1] Siegel R, Ma J, Zou Z, Jemal A 2014 Cancer statistics, 2014. CA Cancer J. Clin. 64, 9–29. (doi:10.3322/caac.21208) 24399786 10.3322/caac.21208

[2] TCGA 2011 Integrated genomic analyses of ovarian carcinoma. Nature 474, 609–615. (doi:10.1038/nature10166) 21720365 10.1038/nature10166 PMC3163504

[3] Pan Y et al. 2016 miR-509-3p is clinically significant and strongly attenuates cellular migration and multi-cellular spheroids in ovarian cancer. Oncotarget 7, 25 930–25 948. (doi:10.18632/oncotarget.8412) 10.18632/oncotarget.8412 PMC5041955 27036018

[4] Yang C, Cai J, Wang Q, Tang H, Cao J, Wu L, Wang Z 2012 Epigenetic silencing of miR-130b in ovarian cancer promotes the development of multidrug resistance by targeting colony-stimulating factor 1. Gynecol. Oncol. 124, 325–334. (doi:10.1016/j.ygyno.2011.10.013) 22005523 10.1016/j.ygyno.2011.10.013

[5] Zhai Q, Zhou L, Zhao C, Wan J, Yu Z, Guo X, Qin J, Chen J, Lu R 2012 Identification of miR-508-3p and miR-509-3p that are associated with cell invasion and migration and involved in apoptosis of renal cell carcinoma. Biochem. Biophys. Res. Commun. 419, 621–626. (doi:10.1016/j.bbrc.2012.02.060) 22369946 10.1016/j.bbrc.2012.02.060

[6] Yoon S, Han E, Choi Y-C, Kee H, Jeong Y, Yoon J, Baek K 2014 Inhibition of cell proliferation and migration by miR-509-3p that targets CDK2, Rac1, and PIK3C2A. Mol. Cells 37, 314–321. (doi:10.14348/molcells.2014.2360) 24802056 10.14348/molcells.2014.2360 PMC4012080

[7] Hanahan D, Weinberg RA 2011 Hallmarks of cancer: the next generation. Cell 144, 646–674. (doi:10.1016/j.cell.2011.02.013) 21376230 10.1016/j.cell.2011.02.013

[8] Agus DB et al. 2013 A physical sciences network characterization of non-tumorigenic and metastatic cells. Sci. Rep. 3, 1449 (doi:10.1038/srep01449) 23618955 10.1038/srep01449 PMC3636513

[9] Mierke CT 2013 The role of focal adhesion kinase in the regulation of cellular mechanical properties. Phys. Biol. 10, 065005 (doi:10.1088/1478-3975/10/6/065005) 24304934 10.1088/1478-3975/10/6/065005

[10] Lee GYH, Lim CT 2007 Biomechanics approaches to studying human diseases. Trends Biotechnol. 25, 111–118. (doi:10.1016/j.tibtech.2007.01.005) 17257698 10.1016/j.tibtech.2007.01.005

[11] Mierke CT 2013 Physical break-down of the classical view on cancer cell invasion and metastasis. Eur. J. Cell Biol. 92, 89–104. (doi:10.1016/j.ejcb.2012.12.002) 23391781 10.1016/j.ejcb.2012.12.002

[12] Frantz C, Stewart KM, Weaver VM 2010 The extracellular matrix at a glance. J. Cell Sci. 123, 4195–4200. (doi:10.1242/jcs.023820) 21123617 10.1242/jcs.023820 PMC2995612

[13] Wolf K, Friedl P 2011 Extracellular matrix determinants of proteolytic and non-proteolytic cell migration. Trends Cell Biol. 21, 736–744. (doi:10.1016/j.tcb.2011.09.006) 22036198 10.1016/j.tcb.2011.09.006

[14] Weigelin B, Bakker G, Friedl P 2012 Intravital third harmonic generation microscopy of collective melanoma cell invasion. Intravital 1, 32–43. (doi:10.4161/intv.21223) 10.4161/intv.21223 PMC5858865 29607252

[15] Yurchenco PD, Ruben GC 1987 Basement-membrane structure *in situ*—evidence for lateral associations in the type-IV collagen network. J. Cell Biol. 105, 2559–2568. (doi:10.1083/jcb.105.6.2559) 3693393 10.1083/jcb.105.6.2559 PMC2114722

[16] Lautscham L et al. 2015 Migration in confined 3D environments is determined by a combination of adhesiveness, nuclear volume, contractility, and cell stiffness. Biophys. J. 109, 900–913. (doi:10.1016/j.bpj.2015.07.025) 26331248 10.1016/j.bpj.2015.07.025 PMC4564685

[17] Zaman MH, Trapani LM, Sieminski AL, MacKellar D, Gong H, Kamm RD, Wells A, Lauffenburger DA, Matsudaira P 2006 Migration of tumor cells in 3D matrices is governed by matrix stiffness along with cell-matrix adhesion and proteolysis. Proc. Natl Acad. Sci. USA 103, 10 889–10 894. (doi:10.1073/pnas.0604460103) 16832052 10.1073/pnas.0604460103 PMC1544144

[18] Wolf K, Mazo I, Leung H, Engelke K, von Andrian UH, Deryugina EI, Strongin AY, Bröcker E-B, Friedl P 2003 Compensation mechanism in tumor cell migration: mesenchymal-amoeboid transition after blocking of pericellular proteolysis. J. Cell Biol. 160, 267–277. (doi:10.1083/jcb.200209006) 12527751 10.1083/jcb.200209006 PMC2172637

[19] Wolf K, Wu YI, Liu Y, Geiger J, Tam E, Overall C, Stack MS, Friedl P 2007 Multi-step pericellular proteolysis controls the transition from individual to collective cancer cell invasion. Nat. Cell Biol. 9, 893–904. (doi:10.1038/ncb1616) 17618273 10.1038/ncb1616

[20] Suresh S 2007 Biomechanics and biophysics of cancer cells. Acta Biomater. 3, 413–438. (doi:10.1016/j.actbio.2007.04.002) 17540628 10.1016/j.actbio.2007.04.002 PMC2917191

[21] Cross SE, Jin Y-S, Rao J, Gimzewski JK 2007 Nanomechanical analysis of cells from cancer patients. Nat. Nanotechnol. 2, 780–783. (doi:10.1038/nnano.2007.388) 18654431 10.1038/nnano.2007.388

[22] Guck J et al. 2005 Optical deformability as an inherent cell marker for testing malignant transformation and metastatic competence. Biophys. J. 88, 3689–3698. (doi:10.1529/biophysj.104.045476) 15722433 10.1529/biophysj.104.045476 PMC1305515

[23] Xu W, Mezencev R, Kim B, Wang L, McDonald J, Sulchek T 2012 Cell stiffness is a biomarker of the metastatic potential of ovarian cancer cells. PLoS ONE 7, e46609 (doi:10.1371/journal.pone.0046609) 23056368 10.1371/journal.pone.0046609 PMC3464294

[24] Swaminathan V, Mythreye K, O'Brien ET, Berchuck A, Blobe GC, Superfine R 2011 Mechanical stiffness grades metastatic potential in patient tumor cells and in cancer cell lines. Cancer Res. 71, 5075–5080. (doi:10.1158/0008-5472.CAN-11-0247) 21642375 10.1158/0008-5472.CAN-11-0247 PMC3220953

[25] Hoelzle DJ, Varghese BA, Chan CK, Rowat AC 2014 A microfluidic technique to probe cell deformability. J. Vis. Exp. 91, e51474 (doi:10.3791/51474) 10.3791/51474 PMC4828024 25226269

[26] Nyberg KD, Scott MB, Bruce SL, Gopinath AB, Bikos D, Mason TG, Kim JW, Choi HS, Rowat AC 2016 The physical origins of transit time measurements for rapid, single cell mechanotyping. Lab Chip 16, 3330–3339. (doi:10.1039/C6LC00169F) 27435631 10.1039/c6lc00169f

[27] Qi D, Kaur Gill N, Santiskulvong C, Sifuentes J, Dorigo O, Rao J, Taylor-Harding B, Ruprecht Wiedemeyer W, Rowat AC 2015 Screening cell mechanotype by parallel microfiltration. Sci. Rep. 5, 17595 (doi:10.1038/srep17595) 26626154 10.1038/srep17595 PMC4667223

[28] Hou HW, Li QS, Lee GYH, Kumar AP, Ong CN, Lim CT 2009 Deformability study of breast cancer cells using microfluidics. Biomed. Microdevices 11, 557–564. (doi:10.1007/s10544-008-9262-8) 19082733 10.1007/s10544-008-9262-8

[29] Byun S, Son S, Amodei D, Cermak N, Shaw J, Ho J, Hecht VC 2013 Characterizing deformability and surface friction of cancer cells. Proc. Natl Acad. Sci. USA 110, 7580–7585. (doi:10.1073/pnas.1218806110) 23610435 10.1073/pnas.1218806110 PMC3651488

[30] Rosenbluth MJ, Lam WA, Fletcher DA 2008 Analyzing cell mechanics in hematologic diseases with microfluidic biophysical flow cytometry. Lab Chip 8, 1062–1070. (doi:10.1039/b802931h) 18584080 10.1039/b802931h PMC7931849

[31] Adamo A, Sharei A, Adamo L, Lee B, Mao S, Jensen KF 2012 Microfluidics-based assessment of cell deformability. Anal. Chem. 84, 6438–6443. (doi:10.1021/ac300264v) 22746217 10.1021/ac300264v PMC3418411

[32] Gunaratne et al. Submitted Tumor-targeted delivery of microRNA-130b, a novel therapeutic strategy for circumventing mutations in p53 by inducing family member TAp63.

[33] Yu X et al. 2013 MiRNA expression signature for potentially predicting the prognosis of ovarian serous carcinoma. Tumour Biol. 34, 3501–3508. (doi:10.1007/s13277-013-0928-3) 23836287 10.1007/s13277-013-0928-3

[34] Vang S et al. 2013 Identification of ovarian cancer metastatic miRNAs. PLoS ONE 8, e58226 (doi:10.1371/journal.pone.0058226) 23554878 10.1371/journal.pone.0058226 PMC3595263

[35] Cho A, Howell VM, Colvin EK 2015 The extracellular matrix in epithelial ovarian cancer—a piece of a puzzle. Front. Oncol. 5, 1–16. (doi:10.3389/fonc.2015.00245) 26579497 10.3389/fonc.2015.00245 PMC4629462

[36] Hu X, Li D, Zhang W, Zhou J, Tang B, Li L 2012 Matrix metalloproteinase-9 expression correlates with prognosis and involved in ovarian cancer cell invasion. Arch. Gynecol. Obstet. 286, 1537–1543. (doi:10.1007/s00404-012-2456-6) 22832979 10.1007/s00404-012-2456-6

[37] Davidson B, Goldberg I, Gotlieb WH, Kopolovic J, Ben-Baruch G, Nesland JM, Berner A, Bryne M, Reich R 1999 High levels of MMP-2, MMP-9, MT1-MMP and TIMP-2 mRNA correlate with poor survival in ovarian carcinoma. Clin. Exp. Metastasis 17, 799–808. (doi:10.1023/A:1006723011835) 11089877 10.1023/a:1006723011835

[38] Huang S, Van Arsdall M, Tedjarati S, McCarty M, Wu W, Langley R, Fidler IJ 2002 Contributions of stromal metalloproteinase-9 to angiogenesis and growth of human ovarian carcinoma in mice. J. Natl. Cancer Inst. 94, 1134–1142. (doi:10.1093/jnci/94.15.1134) 12165638 10.1093/jnci/94.15.1134

[39] Sodek KL, Brown TJ, Ringuette MJ 2008 Collagen I but not Matrigel matrices provide an MMP-dependent barrier to ovarian cancer cell penetration. BMC Cancer 8, 1–11. (doi:10.1186/1471-2407-8-223) 18681958 10.1186/1471-2407-8-223 PMC2519089

[40] Miow QH, Tan TZ, Ye J, Lau JA, Yokomizo T, Thiery J, Mori S 2015 Epithelial–mesenchymal status renders differential responses to cisplatin in ovarian cancer. Oncogene 34, 1899–1907. (doi:10.1038/onc.2014.136) 24858042 10.1038/onc.2014.136

[41] Huang T et al. 2016 miR-508-3p concordantly silences NFKB1 and RELA to inactivate canonical NF-κB signaling in gastric carcinogenesis. Mol. Cancer 15, 9 (doi:10.1186/s12943-016-0493-7) 26801246 10.1186/s12943-016-0493-7 PMC4724081

[42] Zhao G et al. 2013 MiR-130b is a prognostic marker and inhibits cell proliferation and invasion in pancreatic cancer through targeting STAT3. PLoS ONE 8, e73803 (doi:10.1371/journal.pone.0073803) 24040078 10.1371/journal.pone.0073803 PMC3769379

[43] Chan CJ, Ekpenyong AE, Golfier S, Li W, Chalut KJ, Otto O, Elgeti J, Guck J, Lautenschläger F 2015 Myosin II activity softens cells in suspension. Biophys. J. 108, 1856–1869. (doi:10.1016/j.bpj.2015.03.009) 25902426 10.1016/j.bpj.2015.03.009 PMC4407259

[44] Lange JR, Steinwachs J, Kolb T, Lautscham LA, Harder I, Whyte G, Fabry B 2015 Microconstriction arrays for high-throughput quantitative measurements of cell mechanical properties. Biophys. J. 109, 26–34. (doi:10.1016/j.bpj.2015.05.029) 26153699 10.1016/j.bpj.2015.05.029 PMC4571004

[45] Wang G, Mao W, Byler R, Patel K, Henegar C, Alexeev A, Sulchek T 2013 Stiffness dependent separation of cells in a microfluidic device. PLoS ONE 8, e75901 (doi:10.1371/journal.pone.0075901) 24146787 10.1371/journal.pone.0075901 PMC3797716

[46] Fan X, Kurgan L 2014 Comprehensive overview and assessment of computational prediction of microRNA targets in animals. Brief. Bioinform. 16, 780–794. (doi:10.1093/bib/bbu044) 25471818 10.1093/bib/bbu044

[47] Prendergast GC 2001 Actin’ up: RhoB in cancer and apoptosis. Nat. Rev. Cancer 1, 162–168. (doi:10.1038/35101096) 11905808 10.1038/35101096

[48] Ridley AJ 2013 RhoA, RhoB and RhoC have different roles in cancer cell migration. J. Microsc. 251, 242–249. (doi:10.1111/jmi.12025) 23488932 10.1111/jmi.12025

[49] Wheeler AP, Ridley AJ 2004 Why three Rho proteins? RhoA, RhoB, RhoC, and cell motility. Exp. Cell Res. 301, 43–49. (doi:10.1016/j.yexcr.2004.08.012) 15501444 10.1016/j.yexcr.2004.08.012

[50] Maeda M et al. 2011 ARHGAP18, a GTPase-activating protein for RhoA, controls cell shape, spreading, and motility. Mol. Biol. Cell 22, 3840–3852. (doi:10.1091/mbc.E11-04-0364) 21865595 10.1091/mbc.E11-04-0364 PMC3192863

[51] Kustermans G, El Mjiyad N, Horion J, Jacobs N, Piette J, Legrand-Poels S 2008 Actin cytoskeleton differentially modulates NF-κB-mediated IL-8 expression in myelomonocytic cells. Biochem. Pharmacol. 76, 1214–1228. (doi:10.1016/j.bcp.2008.08.017) 18789311 10.1016/j.bcp.2008.08.017

[52] Nemeth ZH, Deitch EA, Davidson MT, Szabo C, Vizi ES, Hasko G 2004 Disruption of the actin cytoskeleton results in nuclear factor-κB activation and inflammatory mediator production in cultured human intestinal epithelial cells. J. Cell. Physiol. 200, 71–81. (doi:10.1002/jcp.10477) 15137059 10.1002/jcp.10477

[53] Becker-Weimann S, Xiong G, Furuta S, Han J, Kuhn I, Akavia U-D, Pe'er D, Bissell MJ, Xu R 2013 NFκB disrupts tissue polarity in 3D by preventing integration of microenvironmental signals. Oncotarget 4, 2010–2020. (doi:10.18632/oncotarget.1451) 24243820 10.18632/oncotarget.1451 PMC3875766

[54] Dierks S, Hardenberg S, Von, Schmidt T, Bremmer F, Burfeind P, Kaulfuß S 2015 Leupaxin stimulates adhesion and migration of prostate cancer cells through modulation of the phosphorylation status of the actin-binding protein caldesmon. Oncotarget 6, 13 591–13 606. (doi:10.18632/oncotarget.3792) 10.18632/oncotarget.3792 PMC4537036 26079947

[55] Chen PW, Kroog GS 2010 Leupaxin is similar to paxillin in focal adhesion targeting and tyrosine phosphorylation but has distinct roles in cell adhesion and spreading. Cell Adhes. Migr. 4, 527–540. (doi:10.4161/cam.4.4.12399) 10.4161/cam.4.4.12399 PMC3011259 20543562

[56] Pollard TD 2007 Regulation of actin filament assembly by Arp2/3 complex and formins. Annu. Rev. Biophys. Biomol. Struct. 36, 451–477. (doi:10.1146/annurev.biophys.35.040405.101936) 17477841 10.1146/annurev.biophys.35.040405.101936

[57] Porazinski S et al. 2015 YAP is essential for tissue tension to ensure vertebrate 3D body shape. Nature 521, 217–221. (doi:10.1530/ERC-14-0411.Persistent) 25778702 10.1038/nature14215 PMC4720436

[58] Chang GHK, Lay AJ, Ting KK, Gamble JR 2014 ARHGAP18: an endogenous inhibitor of angiogenesis, limiting tip formation and stabilizing junctions. Small GTPases 5, e975002 (doi:10.4161/21541248.2014.975002) 10.4161/21541248.2014.975002 PMC4601187 25425145

[59] Sit S-T, Manser E 2011 Rho GTPases and their role in organizing the actin cytoskeleton. J. Cell Sci. 124, 679–683. (doi:10.1242/jcs.064964) 21321325 10.1242/jcs.064964

[60] Vial E, Sahai E, Marshall CJ 2003 ERK-MAPK signaling coordinately regulates activity of Rac1 and RhoA for tumor cell motility. Cancer Cell 4, 67–79. (doi:10.1016/S1535-6108(03)00162-4) 12892714 10.1016/s1535-6108(03)00162-4

[61] Ridley AJ 2001 Rho GTPases and cell migration. J. Cell Sci. 114, 2713–2722. (doi:10.1083/jcb.150.4.807) 11683406 10.1242/jcs.114.15.2713

[62] Mack NA, Whalley HJ, Castillo-Lluva S, Malliri A 2011 The diverse roles of Rac signaling in tumorigenesis. Cell Cycle 10, 1571–1581. (doi:10.4161/cc.10.10.15612) 21478669 10.4161/cc.10.10.15612 PMC3127158

[63] Ahn Y et al. 2012 ZEB1 drives prometastatic actin cytoskeletal remodeling by downregulating miR-34a expression. J. Clin. Invest. 122, 3170–3183. (doi:10.1172/JCI63608DS1) 22850877 10.1172/JCI63608 PMC3428095

[64] Mendez M, Kojima S, Goldman RD 2010 Vimentin induces changes in cell shape, motility, and adhesion during the epithelial to mesenchymal transition. FASEB J. 24, 1838–1851. (doi:10.1096/fj.09-151639) 20097873 10.1096/fj.09-151639 PMC2874471

[65] Liu C, Lin H, Tang M, Wang Y 2015 Vimentin contributes to epithelial-mesenchymal transition cancer cell mechanics by mediating cytoskeletal organization and focal adhesion maturation. Oncotarget 6, 15 966–15 983. (doi:10.18632/oncotarget.3862) 10.18632/oncotarget.3862 PMC4599250 25965826

[66] Wang N, Stamenović D 2000 Contribution of intermediate filaments to cell stiffness, stiffening, and growth. Am. J. Physiol. Cell Physiol. 279, C188–C194. (doi:10.1109/IEMBS.1999.802102) 10898730 10.1152/ajpcell.2000.279.1.C188

[67] Eckes B et al. 1998 Impaired mechanical stability, migration and contractile capacity in vimentin-deficient fibroblasts. J. Cell Sci. 111, 1897–1907. 9625752 10.1242/jcs.111.13.1897

[68] Karlsson R, Pedersen ED, Wang Z, Brakebusch C 2009 Rho GTPase function in tumorigenesis. Biochim. Biophys. Acta 1796, 91–98. (doi:10.1016/j.bbcan.2009.03.003) 19327386 10.1016/j.bbcan.2009.03.003

[69] Wu M, Wu ZF, Kumar-Sinha C, Chinnaiyan A, Merajver SD 2004 RhoC induces differential expression of genes involved in invasion and metastasis in MCF10A breast cells. Breast Cancer Res. Treat. 84, 3–12. (doi:10.1023/B:BREA.0000018426.76893.21) 14999149 10.1023/B:BREA.0000018426.76893.21

[70] Yu T, Cao R, Li S, Fu M, Ren L, Chen W, Zhu H, Zhan Q, Shi R 2015 MiR-130b plays an oncogenic role by repressing PTEN expression in esophageal squamous cell carcinoma cells. BMC Cancer 15, 1–9. (doi:10.1186/s12885-015-1031-5) 25637514 10.1186/s12885-015-1031-5 PMC4318221

[71] Truong LD, Dryer SE, Hu Z, Xu J 2015 Loss of PTEN promotes podocyte cytoskeletal rearrangement, aggravating diabetic nephropathy. J. Pathol. 236, 30–40. (doi:10.1002/path.4508) 25641678 10.1002/path.4508 PMC4398628

[72] Liliental J, Moon SY, Lesche R, Mamillapalli R, Li D, Zheng Y, Sun H, Wu H 2000 Genetic deletion of the Pten tumor suppressor gene promotes cell motility by activation of Rac1 and Cdc42 GTPases. Curr. Biol. 10, 401–404. (doi:10.1016/S0960-9822(00)00417-6) 10753747 10.1016/s0960-9822(00)00417-6

[73] Romero S, Le Clainche C, Didry D, Egile C, Pantaloni D, Carlier MF 2004 Formin is a processive motor that requires profilin to accelerate actin assembly and associated ATP hydrolysis. Cell 119, 419–429. (doi:10.1016/j.cell.2004.09.039) 15507212 10.1016/j.cell.2004.09.039

[74] Kovar DR, Harris ES, Mahaffy R, Higgs HN, Pollard TD 2006 Control of the assembly of ATP- and ADP-actin by formins and profilin. Cell 124, 423–435. (doi:10.1016/j.cell.2005.11.038) 16439214 10.1016/j.cell.2005.11.038

[75] Peng J, Wallar BJ, Flanders A, Swiatek PJ, Alberts AS 2003 Disruption of the Diaphanous-related formin Drf1 gene encoding mDia1 reveals a role for Drf3 as an effector for Cdc42. Curr. Biol. 13, 534–545. (doi:10.1016/S0960-9822(03)00170-2) 12676083 10.1016/s0960-9822(03)00170-2

[76] Stastna J, Pan X, Wang H, Kollmannsperger A, Kutscheidt S, Lohmann V, Grosse R, Fackler OT 2012 Differing and isoform-specific roles for the formin DIAPH3 in plasma membrane blebbing and filopodia formation. Cell Res. 22, 728–745. (doi:10.1038/cr.2011.202) 22184005 10.1038/cr.2011.202 PMC3317560

[77] Campellone KG, Welch MD 2010 A nucleator arms race: cellular control of actin assembly. Nat. Rev. Mol. Cell Biol. 11, 237–251. (doi:10.1038/nrm2867) 20237478 10.1038/nrm2867 PMC2929822

[78] Kühn S, Geyer M 2014 Formins as effector proteins of Rho GTPases. Small GTPases 5, e29513 (doi:10.4161/sgtp.29513) 24914801 10.4161/sgtp.29513 PMC4111664

[79] Nakamura F, Hartwig JH, Stossel TP, Szymanski PT 2005 Ca^2+^ and calmodulin regulate the binding of filamin A to actin filaments. J. Biol. Chem. 280, 32 426–32 433. (doi:10.1074/jbc.M502203200) 10.1074/jbc.M502203200 16030015

[80] Ridley AJ, Hall A 1992 The small GTP-binding protein rho regulates the assembly of focal adhesions and actin stress fibers in response to growth factors. Cell 70, 389–399. (doi:10.1016/0092-8674(92)90163-7) 1643657 10.1016/0092-8674(92)90163-7

[81] Amano M, Chihara K, Kimura K, Fukata Y, Nakamura N, Matsuura Y, Kaibuchi K 1997 Formation of actin stress fibers and focal adhesions enhanced by Rho-kinase formation of actin stress fibers and focal adhesions enhanced by Rho-kinase. Science 275, 1308–1311. (doi:10.1126/science.275.5304.1308) 9036856 10.1126/science.275.5304.1308

[82] Comer KA, Dennis PA, Armstrong L, Catino JJ, Kastan MB, Kumar CC 1998 Human smooth muscle alpha-actin gene is a transcriptional target of the p53 tumor suppressor protein. Oncogene 16, 1299–1308. (doi:10.1038/sj.onc.1201645) 9546431 10.1038/sj.onc.1201645

[83] Rønnov-Jessen L, Petersen OW 1996 A function for filamentous α-smooth muscle actin: retardation of motility in fibroblasts. J. Cell Biol. 134, 67–80. (doi:10.1083/jcb.134.1.67) 8698823 10.1083/jcb.134.1.67 PMC2120928

[84] Sarrió D, Rodriguez-Pinilla SM, Hardisson D, Cano A, Moreno-Bueno G, Palacios J 2008 Epithelial-mesenchymal transition in breast cancer relates to the basal-like phenotype. Cancer Res. 68, 989–997. (doi:10.1158/0008-5472.CAN-07-2017) 18281472 10.1158/0008-5472.CAN-07-2017

[85] Lee HW et al. 2013 Alpha-smooth muscle actin (ACTA2) is required for metastatic potential of human lung adenocarcinoma. Clin. Cancer Res. 19, 5879–5889. (doi:10.1158/1078-0432.CCR-13-1181) 23995859 10.1158/1078-0432.CCR-13-1181

[86] Peckham M, Miller G, Wells C, Zicha D, Dunn GA 2001 Specific changes to the mechanism of cell locomotion induced by overexpression of (β)-actin. J. Cell Sci. 114, 1367–1377. 11257002 10.1242/jcs.114.7.1367

[87] Dugina V, Zwaenepoel I, Gabbiani G, Clément S, Chaponnier C 2009 Beta and gamma-cytoplasmic actins display distinct distribution and functional diversity. J. Cell Sci. 122, 2980–2988. (doi:10.1242/jcs.041970) 19638415 10.1242/jcs.041970

[88] Guo M, Ehrlicher AJ, Mahammad S, Fabich H, Jensen MH, Moore JR, Fredberg JJ, Goldman RD, Weitz DA 2013 The role of vimentin intermediate filaments in cortical and cytoplasmic mechanics. Biophys. J. 105, 1562–1568. (doi:10.1016/j.bpj.2013.08.037) 24094397 10.1016/j.bpj.2013.08.037 PMC3791300

[89] Janmey PA, Euteneuer U, Traub P, Schliwa M 1991 Viscoelastic properties of vimentin compared with other filamentous biopolymer networks. J. Cell Biol. 113, 155–160. (doi:10.1083/jcb.113.1.155) 2007620 10.1083/jcb.113.1.155 PMC2288924

[90] Wiche G, Winter L 2011 Plectin isoforms as organizers of intermediate filament cytoarchitecture. Bioarchitecture 1, 14–20. (doi:10.4161/bioa.1.1.14630) 21866256 10.4161/bioa.1.1.14630 PMC3158638

[91] Wiche G 1998 Role of plectin in cytoskeleton organization and dynamics. J. Cell Sci. 111, 2477–2486. (doi:10.1096/fj.08-124453) 9701547 10.1242/jcs.111.17.2477

[92] Svitkina TM, Verkhovsky AB, Borisy GG 1996 Plectin sidearms mediate interaction of intermediate filaments with microtubules and other components of the cytoskeleton. J. Cell Biol. 135, 991–1007. (doi:10.1083/jcb.135.4.991) 8922382 10.1083/jcb.135.4.991 PMC2133373

[93] Aragona M, Panciera T, Manfrin A, Giulitti S, Michielin F, Elvassore N, Dupont S, Piccolo S 2013 A mechanical checkpoint controls multicellular growth through YAP/TAZ regulation by actin-processing factors. Cell 154, 1047–1059. (doi:10.1016/j.cell.2013.07.042) 23954413 10.1016/j.cell.2013.07.042

[94] Wada K-I, Itoga K, Okano T, Yonemura S, Sasaki H 2011 Hippo pathway regulation by cell morphology and stress fibers. Development 138, 3907–3914. (doi:10.1242/dev.070987) 21831922 10.1242/dev.070987

[95] Dupont S et al. 2011 Role of YAP/TAZ in mechanotransduction. Nature 474, 179–185. (doi:10.1038/nature10137) 21654799 10.1038/nature10137

[96] Piccolo S, Dupont S, Cordenonsi M 2014 The biology of YAP/TAZ: hippo signaling and beyond. Physiol. Rev. 94, 1287–1312. (doi:10.1152/physrev.00005.2014) 25287865 10.1152/physrev.00005.2014

[97] Driscoll TP, Cosgrove BD, Heo S-J, Shurden ZE, Mauck RL 2015 Cytoskeletal to nuclear strain transfer regulates YAP signaling in mesenchymal stem cells. Biophys. J. 108, 2783–2793. (doi:10.1016/j.bpj.2015.05.010) 26083918 10.1016/j.bpj.2015.05.010 PMC4472080

[98] Ketene AN, Roberts PC, Shea AA, Schmelz EM, Agah M 2012 Actin filaments play a primary role for structural integrity and viscoelastic response in cells. Integr. Biol. 4, 540–549. (doi:10.1039/c2ib00168c) 10.1039/c2ib00168c 22446682

[99] Perrin BJ, Ervasti JM 2010 The actin gene family: function follows isoform. Cytoskeleton 67, 630–634. (doi:10.1002/cm.20475) 20737541 10.1002/cm.20475 PMC2949686

[100] Gardel ML, Schneider IC, Aratyn-Schaus Y, Waterman CM 2010 Mechanical integration of actin and adhesion dynamics in cell migration. Annu. Rev. Cell Dev. Biol. 26, 315–333. (doi:10.1146/annurev.cellbio.011209.122036) 19575647 10.1146/annurev.cellbio.011209.122036 PMC4437624

[101] Wirtz D, Konstantopoulos K, Searson PPC 2011 The physics of cancer: the role of physical interactions and mechanical forces in metastasis. Nat. Rev. Cancer 11, 512–522. (doi:10.1038/nrc3080.The) 21701513 10.1038/nrc3080 PMC3262453

[102] Tsai MA, Waugh RE, Keng PC 1998 Passive mechanical behavior of human neutrophils: effects of colchicine and paclitaxel. Biophys. J. 74, 3282–3291. (doi:10.1016/S0006-3495(98)78035-X) 9635782 10.1016/S0006-3495(98)78035-X PMC1299669

[103] Hochmuth RM 2000 Micropipette aspiration of living cells. J. Biomech. 33, 15–22. (doi:10.1016/S0021-9290(99)00175-X) 10609514 10.1016/s0021-9290(99)00175-x

[104] Dong C, Skalak R, Sung K-LP, Schmid-Schönbein GW, Chien S 1988 Passive deformation analysis of human leukocytes. J. Biomech. Eng. 110, 27–36. (doi:10.1115/1.3108402) 3347021 10.1115/1.3108402

[105] Pedersen S, Hoffmann E, Mills J 2001 The cytoskeleton and cell volume regulation. Comp. Biochem. Physiol. Part A 130, 385–399. (doi:10.1016/S1095-6433(01)00429-9) 10.1016/s1095-6433(01)00429-9 11913452

[106] Papakonstanti EA, Vardaki EA, Stournaras C 2000 Actin cytoskeleton: a signaling sensor in cell volume regulation. Cell. Physiol. Biochem. 10, 257–264. (doi:10.1159/000016366) 11125204 10.1159/000016366

[107] Wolf K et al. 2013 Physical limits of cell migration: control by ECM space and nuclear deformation and tuning by proteolysis and traction force. J. Cell Biol. 201, 1069–1084. (doi:10.1083/jcb.201210152) 23798731 10.1083/jcb.201210152 PMC3691458

[108] Reinhart-King CA, Dembo M, Hammer DA 2005 The dynamics and mechanics of endothelial cell spreading. Biophys. J. 89, 676–689. (doi:10.1529/biophysj.104.054320) 15849250 10.1529/biophysj.104.054320 PMC1366566

[109] Califano JP, Reinhart-King CA 2010 Substrate stiffness and cell area predict cellular traction stresses in single cells and cells in contact. Cell. Mol. Bioeng. 3, 68–75. (doi:10.1007/s12195-010-0102-6) 21116436 10.1007/s12195-010-0102-6 PMC2992361

[110] Fu Y, Chin LK, Bourouina T, Liu AQ, VanDongen AMJ 2012 Nuclear deformation during breast cancer cell transmigration. Lab Chip 12, 3774–3778. (doi:10.1039/c2lc40477j) 22864314 10.1039/c2lc40477j

[111] Rowat AC et al. 2013 Nuclear envelope composition determines the ability of neutrophil-type cells to passage through micron-scale constrictions. J. Biol. Chem. 288, 8610–8618. (doi:10.1074/jbc.M112.441535) 23355469 10.1074/jbc.M112.441535 PMC3605679

[112] Davidson PM, Denais C, Bakshi MC, Lammerding J 2014 Nuclear deformability constitutes a rate-limiting step during cell migration in 3-D environments. Cell. Mol. Bioeng. 7, 293–306. (doi:10.1007/s12195-014-0342-y) 25436017 10.1007/s12195-014-0342-y PMC4243304

[113] Denais CM et al. 2016 Nuclear envelope rupture and repair during cancer cell migration. Science 352, 353–358. (doi:10.1126/science.aad7297) 27013428 10.1126/science.aad7297 PMC4833568

[114] Icard-Arcizet D, Cardoso O, Richert A, Hénon S 2008 Cell stiffening in response to external stress is correlated to actin recruitment. Biophys. J. 94, 2906–2913. (doi:10.1529/biophysj.107.118265) 18178644 10.1529/biophysj.107.118265 PMC2267136

[115] Verschueren H, Van der Taelen I, Dewit J, De Braekeleer J, De Baetselier P 1994 Metastatic competence of BW5147 T-lymphoma cell lines is correlated with *in vitro* invasiveness, motility and F-actin content. J. Leukoc. Biol. 55, 552–556. 8145027 10.1002/jlb.55.4.552

[116] Katsantonis J, Tosca A, Koukouritaki SB, Theodoropoulos PA, Gravanis A, Stournaras C 1994 Differences in the G/total actin ratio and microfilament stability between normal and malignant human keratinocytes. Cell Biochem. Funct. 12, 267–274. (doi:10.1002/cbf.290120407) 7834816 10.1002/cbf.290120407

[117] Nürnberg A, Kitzing T, Grosse R 2011 Nucleating actin for invasion. Nat. Rev. Cancer 11, 177–187. (doi:10.1038/nrc3003) 21326322 10.1038/nrc3003

[118] Reichl EM et al. 2008 Interactions between myosin and actin crosslinkers control cytokinesis contractility dynamics and mechanics. Curr. Biol. 18, 471–480. (doi:10.1016/j.cub.2008.02.056) 18372178 10.1016/j.cub.2008.02.056 PMC2361134

[119] Parsons JT, Horwitz AR, Schwartz MA 2010 Cell adhesion: integrating cytoskeletal dynamics and cellular tension. Nat. Rev. Mol. Cell Biol. 11, 633–643. (doi:10.1038/nrm2957) 20729930 10.1038/nrm2957 PMC2992881

[120] Yamaguchi H, Condeelis J 2007 Regulation of the actin cytoskeleton in cancer cell migration and invasion. Biochim. Biophys. Acta 1773, 642–652. (doi:10.1016/j.bbamcr.2006.07.001) 16926057 10.1016/j.bbamcr.2006.07.001 PMC4266238

[121] Petropoulos C et al. 2016 Roles of paxillin family members in adhesion and ECM degradation coupling at invadosomes. J. Cell Biol. 213, 585–599. (doi:10.1083/jcb.201510036) 27269065 10.1083/jcb.201510036 PMC4896053

[122] Gao J et al. 2010 Integrative analysis of complex cancer genomics and clinical profiles using the cBioPortal. Sci. Signal. 6, pl1. (doi:10.1126/scisignal.2004088) 10.1126/scisignal.2004088 PMC4160307 23550210

[123] Cerami E et al. 2012 The cBio cancer genomics portal: an open platform for exploring multidimensional cancer genomics data. Cancer Discov. 2, 401–404. (doi:10.1158/2159-8290.CD-12-0095) 22588877 10.1158/2159-8290.CD-12-0095 PMC3956037

[124] Gen Y et al. 2009 A novel amplification target, ARHGAP5, promotes cell spreading and migration by negatively regulating RhoA in Huh-7 hepatocellular carcinoma cells. Cancer Lett. 275, 27–34. (doi:10.1016/j.canlet.2008.09.036) 18996642 10.1016/j.canlet.2008.09.036

[125] Wong C, Yam JW, Ching Y, Yau T, Leung TH, Jin D, Ng IO 2005 Rho GTPase-activating protein deleted in liver cancer suppresses cell proliferation and invasion in hepatocellular carcinoma. Cancer Res. 65, 8861–8868. (doi:10.1158/0008-5472.CAN-05-1318) 16204057 10.1158/0008-5472.CAN-05-1318

[126] Wang J et al. 2014 ArhGAP30 promotes p53 acetylation and function in colorectal cancer. Nat. Commun. 5, 4735 (doi:10.1038/ncomms5735) 25156493 10.1038/ncomms5735

[127] Luo N, Guo J, Chen L, Yang W, Qu X, Cheng Z 2016 ARHGAP10, downregulated in ovarian cancer, suppresses tumorigenicity of ovarian cancer cells. Cell Death Dis. 7, e2157 (doi:10.1038/cddis.2015.401) 27010858 10.1038/cddis.2015.401 PMC4823924

[128] Fingleton B 2007 Molecular targets in metastasis: lessons from genomic approaches. Cancer Genomics Proteomics 4, 211–222. 17878524

[129] Ueno K, Hirata H, Majid S, Yamamura S, Shahryari V, Tabatabai ZL, Hinoda Y, Dahiya R 2012 Tumor suppressor microRNA-493 decreases cell motility and migration ability in human bladder cancer cells by downregulating RhoC and FZD4. Mol. Cancer Ther. 11, 244–253. (doi:10.1158/1535-7163.MCT-11-0592) 22057916 10.1158/1535-7163.MCT-11-0592 PMC3940358

[130] Jiang L, Liu X, Kolokythas A, Yu J, Wang A, Heidbreder CE, Shi F, Zhou X 2010 Down-regulation of the Rho GTPase signaling pathway is involved in the microRNA-138 mediated inhibition of cell migration and invasion in tongue squamous cell carcinoma. Int. J. Cancer 127, 505–512. (doi:10.1002/ijc.25320.Down-regulation) 20232393 10.1002/ijc.25320 PMC2885137

[131] Morley S et al. 2015 Regulation of microtubule dynamics by DIAPH3 influences amoeboid tumor cell mechanics and sensitivity to taxanes. Sci. Rep. 5, 12136 (doi:10.1038/srep12136) 26179371 10.1038/srep12136 PMC4503992

[132] DeWard AD, Alberts AS 2009 Ubiquitin-mediated degradation of the formin mDia2 upon completion of cell division. J. Biol. Chem. 284, 20 061–20 069. (doi:10.1074/jbc.M109.000885) 10.1074/jbc.M109.000885 PMC2740432 19457867

[133] Creekmore AL, Silkworth WT, Cimini D, Jensen RV, Roberts PC, Schmelz EM 2011 Changes in gene expression and cellular architecture in an ovarian cancer progression model. PLoS ONE 6, e17676 (doi:10.1371/journal.pone.0017676) 21390237 10.1371/journal.pone.0017676 PMC3048403

[134] Pettee KM, Dvorak KM, Nestor-Kalinoski AL, Eisenmann KM 2014 An mDia2/ROCK signaling axis regulates invasive egress from epithelial ovarian cancer spheroids. PLoS ONE 9, e90371 (doi:10.1371/journal.pone.0090371) 24587343 10.1371/journal.pone.0090371 PMC3938721

[135] Calvo F et al. 2013 Mechanotransduction and YAP-dependent matrix remodelling is required for the generation and maintenance of cancer-associated fibroblasts. Nat. Cell Biol. 15, 637–646. (doi:10.1038/ncb2756) 23708000 10.1038/ncb2756 PMC3836234

[136] Secchiero P, Rimondi E, di Iasio MG, Voltan R, Gonelli A, Zauli G 2012 Activation of the p53 pathway induces α-smooth muscle actin expression in both myeloid leukemic cells and normal macrophages. J. Cell. Physiol. 227, 1829–1837. (doi:10.1002/jcp.22910) 21732354 10.1002/jcp.22910

[137] Barretina J et al. 2012 The Cancer Cell Line Encyclopedia enables predictive modelling of anticancer drug sensitivity. Nature 483, 603–607. (doi:10.1038/nature11003) 22460905 10.1038/nature11003 PMC3320027

[138] Fritz G, Kaina B 2001 Ras-related GTPase RhoB represses NF-kB signaling. J. Biol. Chem. 276, 3115–3122. (doi:10.1074/jbc.M005058200) 11062238 10.1074/jbc.M005058200

[139] Domcke S, Sinha R, Levine DA, Sander C, Schultz N 2013 Evaluating cell lines as tumour models by comparison of genomic profiles. Nat. Commun. 4, 2126 (doi:10.1038/ncomms3126) 23839242 10.1038/ncomms3126 PMC3715866

[140] Eitan R et al. 2009 Tumor microRNA expression patterns associated with resistance to platinum based chemotherapy and survival in ovarian cancer patients. Gynecol. Oncol. 114, 253–259. (doi:10.1016/j.ygyno.2009.04.024) 19446316 10.1016/j.ygyno.2009.04.024

[141] Vilming Elgaaen B, Olstad OK, Haug KBF, Brusletto B, Sandvik L, Staff AC, Gautvik KM, Davidson B 2014 Global miRNA expression analysis of serous and clear cell ovarian carcinomas identifies differentially expressed miRNAs including miR-200c-3p as a prognostic marker. BMC Cancer 14, 80 (doi:10.1186/1471-2407-14-80) 24512620 10.1186/1471-2407-14-80 PMC3928323

[142] Chaudhuri O, Koshy ST, Branco da Cunha C, Shin J, Verbeke CS, Allison KH, Mooney DJ 2014 Extracellular matrix stiffness and composition jointly regulate the induction of malignant phenotypes in mammary epithelium. Nat. Mater. 13, 1–35. (doi:10.1038/nmat4009) 24930031 10.1038/nmat4009

[143] Gu Z, Liu F, Tonkova EA, Lee SY, Tschumperlin DJ, Brenner MB 2014 Soft matrix is a natural stimulator for cellular invasiveness. Mol. Biol. Cell 25, 457–469. (doi:10.1091/mbc.E13-05-0260) 24336521 10.1091/mbc.E13-05-0260 PMC3923638

[144] Guzman A, Ziperstein MJ, Kaufman LJ 2014 The effect of fibrillar matrix architecture on tumor cell invasion of physically challenging environments. Biomaterials 35, 6954–6963. (doi:10.1016/j.biomaterials.2014.04.086) 24835043 10.1016/j.biomaterials.2014.04.086

