# Supplementary material for: Tumour-suppressor microRNAs regulate ovarian cancer cell physical properties and invasive behaviour
Source: Open Biol. 2016 Nov 30;6(11):160275. doi: 10.1098/rsob.160275 (PMC5133448; doi:10.1098/rsob.160275)
Supplement: Electronic Supplementary Material [file rsob160275supp1.pdf]

Tumour-suppressor microRNAs regulate ovarian cancer cell physical properties and invasive behaviour

Clara K. Chan[1,2], Yinghong Pan[3], Kendra Nyberg[1,2], Marco A. Marra[6,7], Emilia L. Lim[6], Steven J.M. Jones[6,7,8], Dianna Maar[9], Ewan A. Gibb[6], Preethi H. Gunaratne[3,4,5], A. Gordon Robertson[6], Amy C. Rowat[1,2]

[1] Department of Integrative Biology and Physiology, University of California Los Angeles, Los Angeles, CA. [2] Department of Bioengineering, University of California Los Angeles, Los Angeles, CA. [3] Department of Biochemistry and Biology, University of Houston, Houston, TX. [4] Department of Pathology and Immunology, [5] Human Genome Sequencing Center, Baylor College of Medicine, Houston, TX. [6] Canada's Michael Smith Genome Sciences Centre, British Columbia Cancer Agency, Vancouver, BC, Canada. [7] Department of Medical Genetics, University of British Columbia, Vancouver, BC, Canada. [8] Department of Molecular Biology and Biochemistry, Simon Fraser University, Burnaby, BC, Canada. [9] The Digital Biology Center, Bio-Rad Laboratories, Pleasanton, CA.

Supplementary Material

Supplementary Note

While both PMF and transit time assays require cells to deform through a ~10 μm pore when driven by an external applied pressure, we observe only a moderate correlation ($R = 0.61$, $p = 0.28$) between retention and transit time. This modest correlation between such similar techniques could reflect differences in the readouts for the bulk versus single cell deformability assays. In the microfluidic transit time assay we probe the deformation of single cells through constricted channels over < 1 sec, while in PMF we measure how a population of cells deform through a porous membrane over tens of seconds. The modest

correlation may also stem from differences in the interactions of cells with the polycarbonate membrane filters of our PMF device and the PDMS walls of the microfluidic channels [27–29]. Despite these technical differences between mechanotyping methods, the results of both assays show good agreement.

Supplementary Tables

(a)

Treatment	Median Cell Size	Coefficient of Variation	Kurtosis
**SCR**	20.6	0.2	-2.5
**130b-3p**	23.2	0.2	-2.8
**509-5p**	22.7	0.2	-3.0
**509-3p**	24.4	0.2	-2.9
**508-5p**	22.3	0.2	-3.2
**508-3p**	23.9	0.2	-3.3
**Mock**	21.7	0.2	-2.7

(b)

Treatment	Median Nuclear Size	Coefficient of Variation	Kurtosis
**SCR**	10.0	0.2	8.6
**130b-3p**	11.1	0.2	2.9
**509-5p**	11.1	0.2	2.3
**509-3p**	12.7	0.3	7.4
**508-5p**	11.1	0.2	10.9
**508-3p**	11.6	0.2	2.5
**Mock**	10.0	0.2	15.0

Supplementary Material Table S1. Cell and nuclear size after miR transfection. (a) Summary of median cell size, (b) median nuclear size, coefficient of variation, and kurtosis for miR-treated HEYA8 cells, measured by flow cytometry imaging.

Supplementary Figures

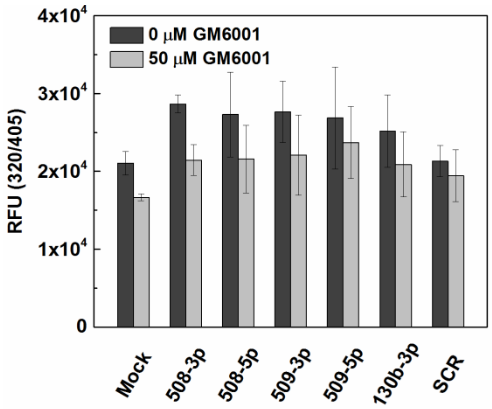

Supplementary Material Figure S1. Effect on protease activity after miRNA overexpression and treatment with the matrix metalloproteinase inhibitor GM6001. Quantification of protease activity in cell culture conditioned media measured using a fluorogenic peptide substrate (N=2). Relative fluorescence units (RFU) are obtained at 320 nm excitation and 405 nm emission. The background fluorescence is subtracted prior to quantification. Error bars represent standard deviations.

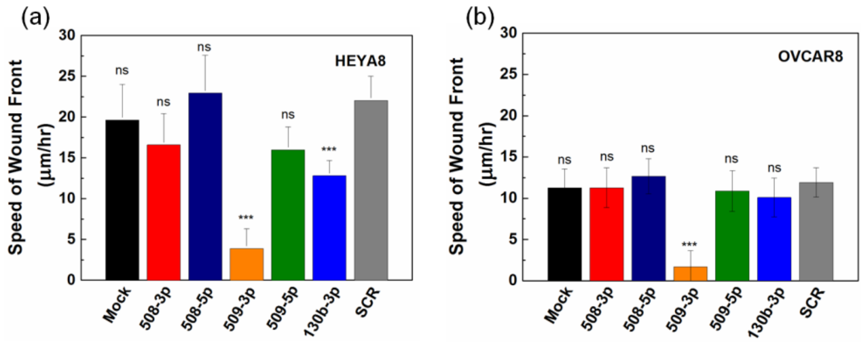

Supplementary Material Figure S2. Effect of miRNA overexpression on cell invasion speed through a collagen matrix. Speed of the wound front (µm/hr) for HEYA8 and OVCAR8 cells, 10 hours after initiation of the scratch wound. The rate of invasion of the ovarian cancer cell lines HEYA8 (~20 µm/hr) and OVCAR8 (~10 µm/hr) is comparable to that of breast cancer cells. In a cell-derived matrix, MDA-MB-231 breast cancer cell migrate through 3D microenvironments at ~0.6 µm/min (~36 µm/hr) [144]. In Matrigel matrices, MDA-MB-231 breast cancer cells migrate at a velocity of ~20 µm/hr [145]. One-way ANOVA with a Tukey post hoc test; *** ($p < 0.001$), ns ($p > 0.05$), compared to the scrambled control treatment (SCR). Error bars show standard deviations from N=3 independent experiments.

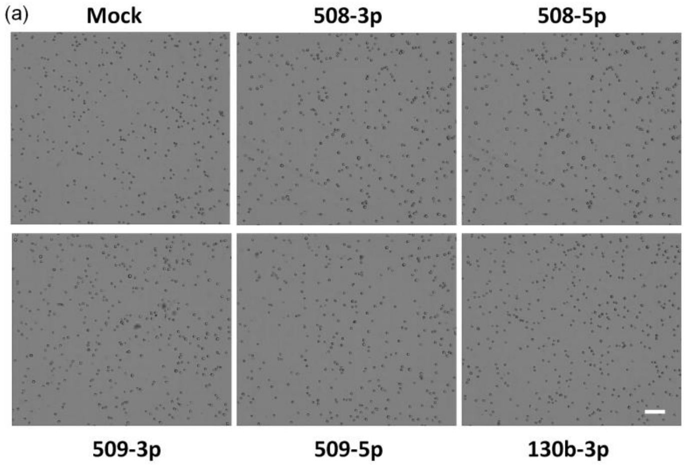

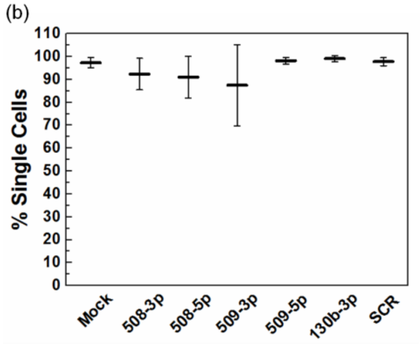

Treatment	% Single Cells
Mock	97.3 ± 2.2
miR-508-3p	92.4 ± 6.9
miR-508-5p	91.0 ± 9.1
miR-509-3p	87.5 ± 17.7
miR-509-5p	98.2 ± 1.4
miR-130b-3p	99.1 ± 1.3
SCR	97.8 ± 1.8

Supplementary Material Figure S3. (a) Images of cell suspensions prior to loading samples into the parallel microfiltration device. Scale, 100 µm. (b) Percentage of single HEYA8 cells in parallel microfiltration samples. Here we identify single cells by manual inspection of the images. Error bars represent standard deviations (N=2).

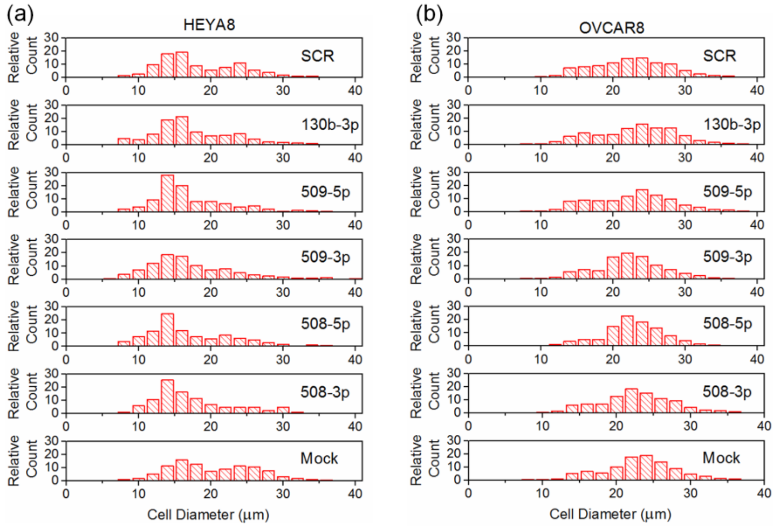

Supplementary Material Figure S4. Cell size after miR transfection. Distribution of cell size for (a) HEYA8 and (b) OVCAR8 cells measured in a microfluidic device prior to entry into the first constriction. As upstream filters can retain cells of larger size, cells imaged in the microfluidic device may appear smaller than those imaged by flow cytometry. Data for 20 to 197 single cells over N=2 independent experiments.

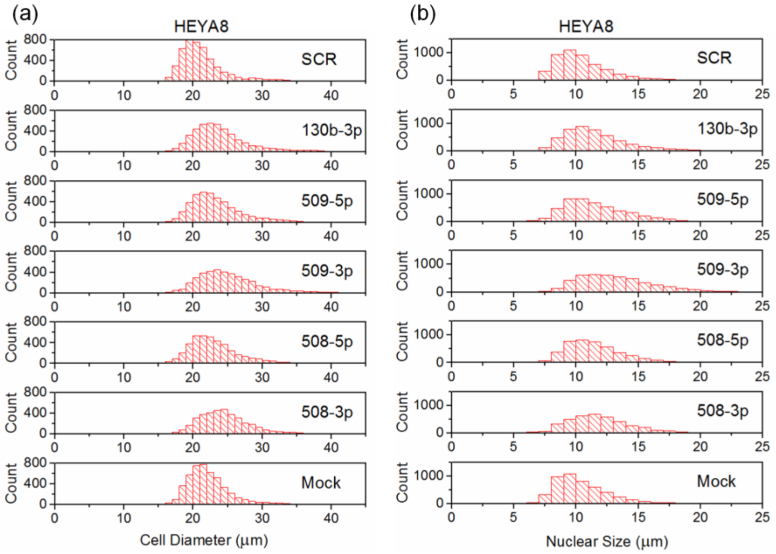

Supplementary Material Figure S5. Cell and nuclear size for HEYA8 after miR transfection. Distribution of (a) cell size and (b) nuclear size measured by flow cytometry imaging. Data for at least 3991 single cells over N=3 independent experiments.

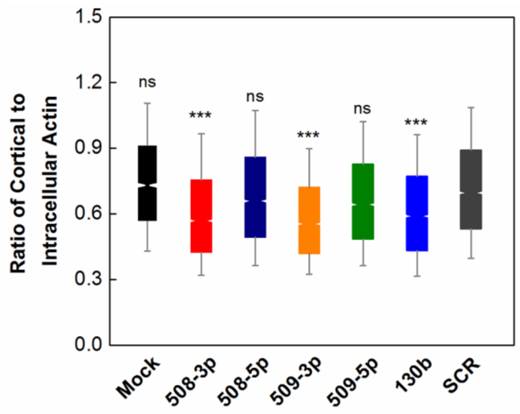

Supplementary Material Figure S6. Subcellular structures of cells in suspension as analyzed by flow cytometer imaging. Quantification of the fluorescence intensity of cortical-to-intracellular F-actin. Data represents n>3991 single cells over N=3 independent experiments. Boxes denote the 25th and 75th percentiles, and whiskers denote the 10th and 90th percentiles. Notches represent the 95% confidence interval about the median. Mann-Whitney test; ns (not significant), *** ($p < 0.001$) compared to the SCR treatment.

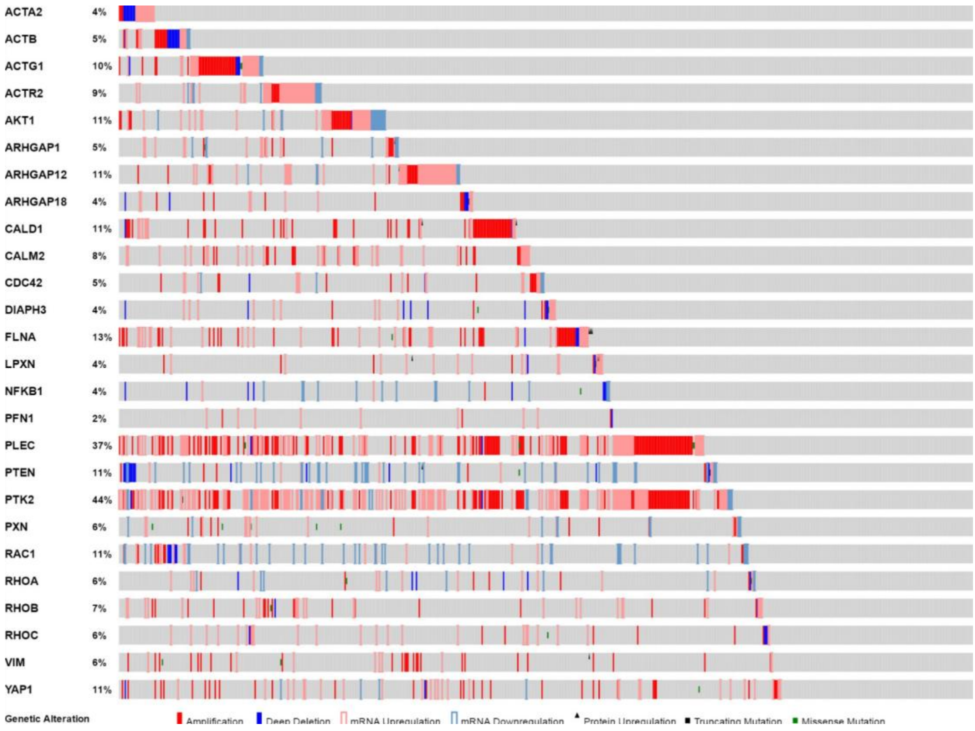

Supplementary Material Figure S7. Analysis of mechanome genes in TCGA ovarian cancer data using cBioPortal. Graphical summary of genomic alterations in mechanome genes across a set of 603 tumour samples (Ovarian serious cystadenocarcinoma, TCGA Provisional; RNA Seq V2 RSEM) [121,122]. Rows represent genes, and columns represent samples. The legend below the graphic indicates the types of alterations.

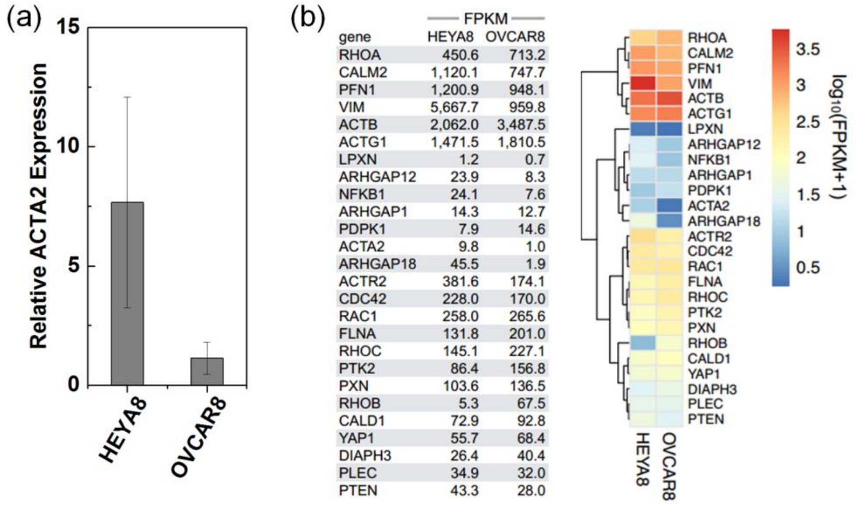

gene	HEYA8 (FPKM)	OVCAR8 (FPKM)
RHOA	450.6	713.2
CALM2	1,120.1	747.7
PFN1	1,200.9	948.1
VIM	5,667.7	959.8
ACTB	2,062.0	3,487.5
ACTG1	1,471.5	1,810.5
LPXN	1.2	0.7
ARHGAP12	23.9	8.3
NFKB1	24.1	7.6
ARHGAP1	14.3	12.7
PDPK1	7.9	14.6
ACTA2	9.8	1.0
ARHGAP18	45.5	1.9
ACTR2	381.6	174.1
CDC42	228.0	170.0
RAC1	258.0	265.6
FLNA	131.8	201.0
RHOC	145.1	227.1
PTK2	86.4	156.8
PXN	103.6	136.5
RHOB	5.3	67.5
CALD1	72.9	92.8
YAP1	55.7	68.4
DIAPH3	26.4	40.4
PLEC	34.9	32.0
PTEN	43.3	28.0

Supplementary Material Figure S8. Analysis of expression of mechanoregulating genes in OVCAR8 and HEYA8 cell lines. (a) Quantification of relative expression as measured by qRT-PCR using the delta delta cycle time method (ΔΔCt) with 18S ribosomal RNA as an endogenous control. Error bars show standard deviations of N=3 independent experiments. (b) Comparison of mRNA levels using RNA-seq data from The Broad-Novartis Cancer Cell Line Encyclopedia (CCLE).
